# 11PS04 is a new chemical entity identified by microRNA-based biosensing with promising therapeutic potential against cancer stem cells

**DOI:** 10.1038/s41598-019-48359-y

**Published:** 2019-08-15

**Authors:** Tania Aguado, José A. Romero-Revilla, Rosario Granados, Susana Campuzano, Rebeca M. Torrente-Rodríguez, Ángel M. Cuesta, Virginia Albiñana, Luisa María Botella, Silvia Santamaría, Jose A. Garcia-Sanz, José Manuel Pingarrón, Francisco Sánchez-Sancho, José-María Sánchez-Puelles

**Affiliations:** 1 Departamento de Biomedicina Molecular, Centro de Investigaciones Biológicas, CSIC, c/Ramiro de Maeztu 9, 28040 Madrid, Spain; 2 Instituto de Química Médica, CSIC, C/Juan de la Cierva 3, 28006 Madrid, Spain; 3 Departamento de Anatomía Patológica, Hospital Universitario de Getafe, Madrid, Spain; 4 Departamento de Química Analítica, Facultad de Ciencias Químicas, Universidad Complutense de Madrid, E-28040 Madrid, Spain

**Keywords:** Molecular medicine, Phenotypic screening

## Abstract

Phenotypic drug discovery must take advantage of the large amount of clinical data currently available. In this sense, the impact of microRNAs (miRs) on human disease and clinical therapeutic responses is becoming increasingly well documented. Accordingly, it might be possible to use miR-based signatures as phenotypic read-outs of pathological status, for example in cancer. Here, we propose to use the information accumulating regarding the biology of miRs from clinical research in the preclinical arena, adapting it to the use of miR biosensors in the earliest steps of drug screening. Thus, we have used an amperometric dual magnetosensor capable of monitoring a miR-21/miR-205 signature to screen for new drugs that restore these miRs to non-tumorigenic levels in cell models of breast cancer and glioblastoma. In this way we have been able to identify a new chemical entity, 11PS04 ((3a*R*,7a*S*)-2-(3-propoxyphenyl)-7,7a-dihydro-3a*H*-pyrano[3,4-*d*]oxazol-6(4*H*)-one), the therapeutic potential of which was suggested in mechanistic assays of disease models, including 3D cell culture (oncospheres) and xenografts. These assays highlighted the potential of this compound to attack cancer stem cells, reducing the growth of breast and glioblastoma tumors *in vivo*. These data demonstrate the enhanced chain of translatability of this strategy, opening up new perspectives for drug-discovery pipelines and highlighting the potential of miR-based electro-analytical sensors as efficient tools in modern drug discovery.

## Introduction

The majority of drug discovery projects currently carried out are target led and while they often identify molecules of interest, they are associated with excessively high rates of attrition when clinical uptake is considered^1–4^. Phenotypic Drug Discovery (PDD) has produced the majority of small-molecule, first-in-class, new molecular entities (NMEs) discovered this century^5^. Accordingly, current PDD programs should endorse enhanced “chains of translatability” to favor the clinical uptake of novel candidate drugs^5,6^. This concept is based on establishing a continuum that links human disease biology to therapeutic activity via the phenotypic elements used for compound screening, a chain that will ultimately determine the success of PDD efforts. Translatability is also enhanced by the use of more appropriate models and culture systems (e.g., 3D cultures), as well as predictive biomarkers^6^. Moreover, the success of PDD approaches relies on combining information regarding the mechanistic basis of the disease with assay readouts, and taking advantage of the ever-increasing clinical data accumulating in virtually all areas of biomedicine^5–8^.

Efforts to discover new cancer drugs have been limited by the failure to apply this concept of “chains of translatability”, partially due to the use of poor predictive tools that are unlikely to fully capture the mosaic nature of tumors^9–11^. Acquired resistance is a common cause of therapeutic failure, often due to the cancer drugs not eliminating small sub-populations of cells in the tumor, such as the cancer stem cells (CSCs). These CSCs represent part of the mosaic that is particularly resistant to radio- and chemo-therapies, and they are currently accepted as a crucial target to improve cancer treatments^9,12–14^. However, since CSCs are generally only a small constituent of tumors, it is not surprising that standard high-throughput 2D cell viability assays on bulk cancer cell populations have generally failed to identify agents with specific CSC toxicity^7,8^.

The past decade has witnessed much interest in the role of microRNAs (miRs) in human pathologies^15,16^, especially in neoplastic diseases^17–19^. MicroRNAs are short non-coding RNAs that repress gene expression post-transcriptionally by targeting the 3′-untranslated region (3′UTR) of specific mRNAs. Indeed, while individual miRs can target multiple mRNAs, it seems that several miRs are likely to be simultaneously involved in the progression of certain diseases^16,19^. Hence, a variety of miR “signatures” have emerged as potential biomarkers, with diagnostic, therapeutic and prognostic value in many human diseases^15^. This is even the case in cancer, where miRs may regulate the expression of oncogenes and tumor suppressor genes. As such, miR-21 has been identified as a proven oncogene and as the only miR overexpressed in a wide variety of cancers, including breast, ovary, cervix, colon, lung, liver, brain, esophagus, prostate and thyroid cancers^18,19^. Moreover, the member of the miR-200 family and miR-205 are key regulators of stem cell fate, proliferation and metastasis, controlling the epithelial–mesenchymal transition (EMT), and miR-205 appears to regulate the proliferation, migration and invasion of breast and glioblastoma (GBM) cells^20–23^.

We recently presented the first disposable amperometric magnetosensor to simultaneously detect miR-21 and miR-205, making it to measure relevant concentrations of both cancer biomarkers^24,25^. The dual amperometric magnetosensor exhibited excellent analytical performance to measure the endogenous levels of both target miRs, without the need for prior amplification, preconcentration or purification. This device combines multiplexed detection and different RNA probes to simultaneously detect multiple miRs. The simple magnetosensor design avoids complicated temperature control, and the relatively low cost of the detection platform opens a new avenue for multi-miR bioanalysis. Indeed, there are many potential applications for such devices in cancer research, not only in clinical diagnosis^24^ but also, as we show here, to search for anti-tumoral compounds that specifically target clinical validated biomarkers.

Here, for the first time we have applied this “minimal miR signature” to drug screening and we developed a PDD strategy with strong predictive validity to identify new anti-tumor drugs that will enhance R&D productivity. As a result, we identified a new chemical entity (NCE), 11PS04 ((3a*R*,7a*S*)-2-(3-propoxyphenyl)-7,7a-dihydro-3a*H*-pyrano[3,4-*d*]oxazol-6(4*H*)-one), that had a demonstrable capacity to combat oncogenicity *in vitro* and *in vivo*, reflecting the predictive value and the enhanced chain of translatability of this PDD strategy based on miR biosensing.

## Results

### Identification of 11PS04 using the dual miR magnetosensor

**Figure 1:**
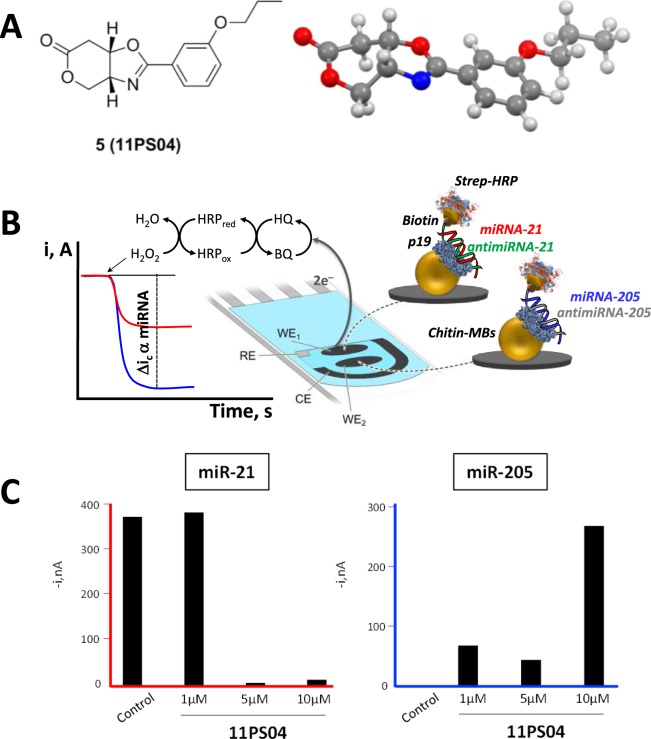
11PS04 modulates the amounts of microRNA-21 and microRNA-205 detected in MCF-7 cells with the dual magnetosensor. (**A**) 2D and 3D structure of compound **5** (**11PS04**), showing the stereochemistry at the ring junction. (**B**) Amperometric traces recorded with the dual magnetosensor reflecting the simultaneous detection of miR-21 and miR-205 in totRNA extracted from MCF-7 cells: Strep-HRP, streptavidin-biotin; p19, viral protein p19; MB, magnetic beads; SPdCE, screen-printed carbon electrodes; HQ, H_2_O_2_/hydroquinone system; WE, working electrode; Δi_c_, variation in cathodic current intensity due to the enzymatic substrate added, indicating proportionality. The 11PS04 structure is shown. (**C**) Detection of miR-21 and miR-205 in totRNA extracted from human breast tissues in the 15 min assay.

Given the undeniable value of quantifying miR-21 and miR-205 expression in models of human cancer, the usefulness of the dual magnetosensor for the simultaneous detection of both target miRs was verified in total RNA extracted from MCF-7 cells exposed to different concentrations of 11PS04 (Fig. 1A–C). The dual magnetosensor was based on the use of a specific viral protein, Carnation Italian ringspot virus (CIRV) p19 protein, that has a high affinity for small double-stranded (ds)RNAs of 19–23 nucleotides in length. This protein was coupled to magnetic microbeads (MBs) as selective magnetocaptors to scavenge the DNA/miRNA heteroduplexes previously formed in solution. These magnetocaptors can be measured by amperometric detection at disposable dual screen-printed carbon electrodes (SPCE), which serve as electrochemical transducers. In brief, the dual magnetosensor relies on efficient hybridization of each target miRNA to a complementary biotinylated RNA probe in solution and the efficient capture of the resulting perfectly matched DNA/miRNA homohybrids by the immobilized CIRV protein. The terminal chitin-binding domain (CBD) of CIRV then binds to the chitin-functionalized magnetic beads (chitin–MBs) that are used as magnetocaptors. After labeling the attached biotinylated RNA homohybrids with a commercial polymer of horseradish peroxidase conjugated streptavidin (Strep-HRP), the resulting MBs bearing the dsRNA/p19complex for each target miRNA were magnetically captured on the corresponding working electrodes (WE1 and WE2) of SPdCEs and amperometric detection was performed at −0.20 V (versus a Ag pseudo reference electrode) in the presence of the H_2_O_2_ (enzyme substrate)/HQ (redox mediator: Fig. 1). The variation in the cathode current measured at each WE, attributed to the enzymatic reduction of H_2_O_2_ mediated by HQ, was proportional to the target miRNA concentration^24,25^. It should be noted that the modification of the MBs is performed individually for each target miRNA in independent eppendorf tubes and that as many MB batches as the determinations required are prepared simultaneously.

MCF-7 cells are known to over-express miR-21 in conjunction with reduced miR-205 expression^25^ and when assayed with the magnetosensor the expected results were obtained from control cultures (Fig. 1). We tested a proprietary compound library of NMEs with unknown mechanisms of action, identifying 11PS04 (3a*R*,7a*S*)-2-(3-propoxyphenyl)-7,7a-dihydro-3a*H*-pyrano[3,4-*d*]oxazol-6(4*H*)-one as a preclinical candidate that clearly reversed the pathological changes in the expression of these two oncomiRs associated with tumorigenesis (Fig. 1, see Supplementary Info (scheme 1) for the chemical synthesis and stereochemical assignment of this compound). Exposing MCF-7 cells for 24 h to concentrations of 5 μM and 10 μM 11PS04 for 24 h virtually eliminated the miR-21 signal, whereas concentrations as low as 1 μM enhanced the signal for miR-205 (Fig. 1C). Hence, this preliminary evaluation indicated that 11PS04 inhibits miR-21 expression while simultaneously enhancing the expression of miR-205, reverting the expression of both oncomiRs to non-tumorigenic ranges. We then determined the range of drug concentrations that did not affect viability of a panel of rodent and human cells, using standard assays (see Supplementary Figs 1 and 2). Significantly, 11PS04 modulated the expression of both miRs at doses that could be considered to be safe (i.e. <10 μM: Fig. 1).

### Validation of the antitumor profile of 11PS04

**Figure 2:**
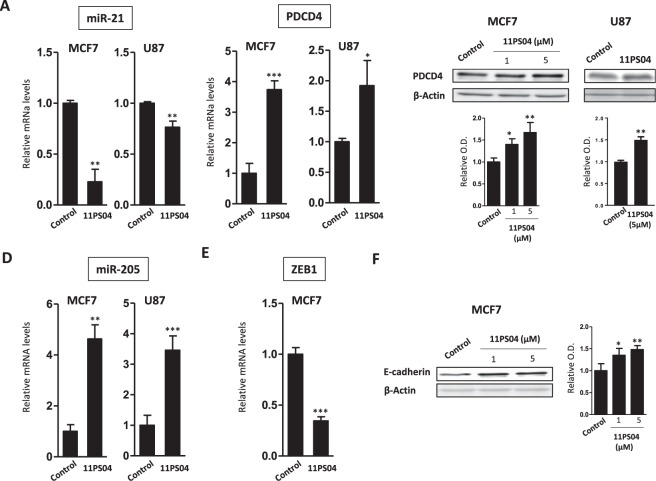
11PS04 regulates the expression of known targets of miR-21 and miR-205 in MCF-7 and U87 cells. Regulation of proteins that are downstream targets of miR-21 and miR-205: PDCD4 and E-Cadherin, respectively. Exposure to 11PS04 (5 μM) led to the transcriptional regulation of miR-21 (**A**) PDCD4 (**B**) miR-205 (**D**) and ZEB1 (**E**). Transcription was compared with the endogenous control: ACTB ribosomal RNA. The accumulation of the miR-21 and miR-205 target proteins in extracts of treated MCF-7 and U87 cells was measured in Western blots, PDCD4 (**C**) and E-Cadherin (**F**) respectively (mean ± SEM; n = 3; *P < 0.05; **P < 0.01; ***P < 0.005 (Student’s t-test).

The read-out of the dual magnetosensor suggest that 11PS04 has the potential to alter the expression of two pivotal miR-biomarkers of breast and GBM cancer. This effect was further validated by quantitative real-time PCR (qPCR: Fig. 2), which confirmed that 11PS04 did indeed inhibit miR-21 and enhance miR-205 expression in a dose-dependent manner. Both these miRs regulate downstream targets relevant to cancer progression and metastasis and thus, the inhibition of miR-21 is likely to produce an upregulation of PDCD4, a protein involved in programmed cell death^26^. Indeed, while the expression of miR-21 was dampened in MCF-7 and U87MG (U87) cells when exposed to a low concentration of 11PS04 for 24 h (5 μM, Fig. 2A), there was an increase in the expression of PDCD4 mRNA and protein (Fig. 2B,C). Furthermore, the increase in miR-205 expression to non-tumorigenic levels after a 24 h exposure to 11PS04 (5 μM) for 24 h in both MCF-7 and U87 cells was correlated with a downregulation of the RNA and protein expression of one of its target genes, the mesenchymal maker involved in EMT and tumor metastasis, ZEB1^20^ (Fig. 2D,E). ZEB1 binds to E-box elements and it represses E-cadherin transcription. Indeed, E-cadherin expression was restored in the presence of 11PS04 as a consequence of ZEB-1 repression (Fig. 2F). Therefore, the changes in miR expression driven by this drug in the two tumor cell lines affect the transcription and translation of their downstream targets. Therefore, these experiments were extended to other tumorigenic cell models of breast cancer (MDA-436) and GBM (C6) cancer, with similar results (see Supplementary Figs 3 and 4).

### 11PS04 restricts the potential of the cancer cell lines to form oncospheres

**Figure 3:**
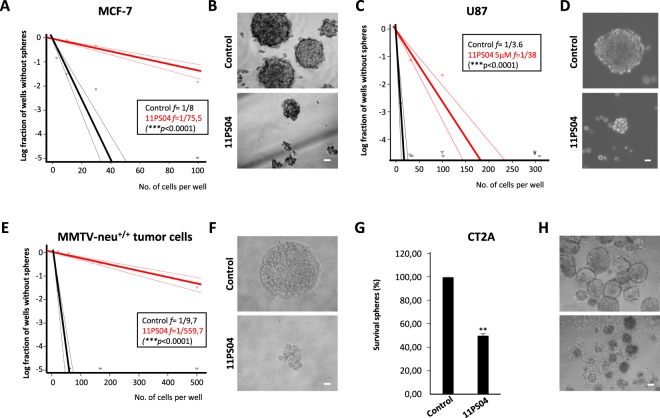
11PS04 impedes sphere formation. The frequency of oncosphere formation was measured by extreme limited dilution analysis (ELDA). (**A**,**C**) Sphere frequencies from MCF-7 and U87 single-cell suspensions are plotted against the number of cells seeded per well, showing a decrease in sphere formation in the presence of 11PS04: solid lines represent the estimated frequency and the broken lines are the 95% confidence intervals. The frequency of initiating sphere formation by cancer stem cells was calculated using the ELDA platform and a significant decrease in the frequency of oncosphere formation was produced by 11PS04 (P value < 0.001). (**B**,**D**) Representative phase-contrast images of mammospheres and gliospheres formed by day 15 (Scale bars 10 and 40 µm). (**E**) The frequencies obtained using mammary gland tumors from MMTV-neu^+/+^ mice under the experimental conditions described in (**A**). (**F**) Representative phase-contrast images of mammospheres from MMTV-neu^+/+^ mice formed by day 15 in the ELDA assay (scale bar 10 µm). (**G**) Relative survival of spheres obtained from CT2A xenograft tumors after 7 days in the presence of 11PS04. (mean ± SEM, n = 3, *P < 0.05, **P < 0.01). (**H**) Representative phase contrast images of the CT2A xenograft tumor spheres (scale bar 100 µm).

Cancer cell lines may contain a subpopulation of CSCs capable of generating non-adherent oncospheres with proliferative potential *in vitro*, a validated procedure to culture CSCs^27–32^. As the capacity to impede the proliferation and development of CSCs is fundamental to the success of anti-cancer therapies, we studied this aspect of the activity of 11PS04. In addition to inhibiting well-accepted biomarkers^33,34^ of “stemness” in breast cancer and glioblastoma (ALDH and Sox-2, respectively: Supplementary Fig. 6), both markers that are associated with a poor prognosis in these two malignancies, we assessed the frequency (*f*) of sphere formation *in vitro* using an extreme limited dilution analysis (ELDA) which indicated the number of tumor cells capable of forming a single sphere^29^. In these experiments, we used safe drug concentrations that do not affect cell viability and we found that such doses of 11PS04 (e.g. <10 μM) significantly suppressed the formation of stem cell spheres in 3D cultures of breast cancer and glioma cancer cells relative to their controls (1/75.5 vs 1/8 in MCF-7 cells, and 1/38 vs 1/3.6 in U87 cells: Fig. 3A–D and Supplementary Fig. 5). Moreover, the generation of secondary 3D colonies from disaggregated primary spheres was impaired even in drug-free medium (control *f* = 1/5.3 vs treated *f* = 1/20: Supplementary Fig. 7).

### 11PS04 abrogates the oncosphere forming potential of cells from the mammary glands of MMTV-neu^+/+^ mice

Breast cancer stem cells from the mammary glands of MMTV-neu^+/+^ mice form oncospheres when cultured in suspension^29^, structures that were also disaggregated by 11PS04 when an ELDA procedure was followed. There were significantly fewer sphere-forming cells when oncospheres were generated in the presence of 11PS04 (5 μM, *p* < 0.001: Fig. 3E,F), and the presence of this compound also impaired the formation of secondary oncospheres from mammary gland cells (Supplementary Fig. 7). 11PS04 did not alter the frequency of secondary mammosphere formation when the contralateral non-tumorigenic mammary gland of the mice was studied as a control (control *f* = 1/31 vs treated *f* = 1/38), indicating that this drug discriminated malignant breast tissue (Supplementary Fig. 7). Moreover, when the glioma CT-2A mouse cell line that presents characteristics of GBM^35,36^, was transplanted subcutaneously to produce CT-2A tumors, well-organized gliospheres were established from disaggregated CT-2A tumors in cultures. The survival of these gliospheres was also significantly compromised on exposure to 11PS04 (5 μM, Fig. 3G,H). In summary, 11PS04 appeared to specifically limit the number of cells with a stem-like phenotype that are capable of generating solid 3D colonies in suspension. This inhibitory activity was most effective when the drug was applied during the period of sphere formation, although it was also capable of acting on well-organized oncospheres.

### 11PS04 treatment delays tumor formation in a mouse xenograft model

**Figure 4:**
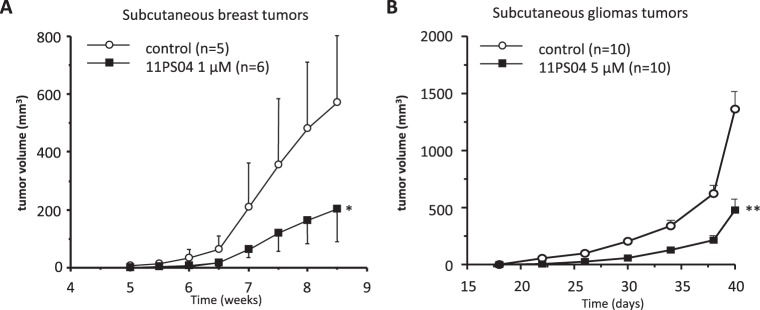
Decreased tumor growth after exposure to 11PS04 cell treatment. (**A**) MDA-MB-436 cells (5 × 10^6^ cells) pre-treated for 48 h with 11PS04 were implanted subcutaneously into immunodeficient Rag2^−/−^ mice. The animals themselves were not treated with the drug. Tumor size was measured weekly and finally, the animals were sacrificed and the tumors extracted. (**B**) NSG immunodepressed mice were inoculated in the flank with 10^6^ 11PS04 treated U-87 glioblastoma cells as xenografts. The tumors were measured from the moment they were large enough to be evaluated.

To study the effect of 11PS04 on xenografts, we measured the tumors formed after subcutaneous injection of MDA-MB-436 human breast cancer cells into the right flank of Rag2^−/−^ mice over 9 weeks. Exposing tumorigenic cells to 11PS04 (1 µM) for 48 h prior to injection significantly restricted tumor growth relative to the cells exposed to the vehicle alone (Fig. 4A), supporting the anti-carcinogenic effect of 11PS04 *in vivo*. To evaluate the *in vivo* relevance of these observation in gliomas, tumor xenografts were generated by subcutaneous injection of U87 cells into 7–8 week-old NOD SCID gamma (NSG) male mice, cells that had been exposed to 11PS04 (5 μM) for 48 h or to the vehicle alone. Importantly, some pre-treated cells were grown *in vitro* in parallel and after 72 h in growth medium, there were no differences in cell viability between cells exposed to 11PS04 or to the vehicle alone (Supplementary Fig. 8A). Strikingly, pre-treatment with 11PS04 decreased the growth of the tumor xenografts relative to the effect produced in the cells exposed to the vehicle alone (Fig. 4B), with a significant delay in the growth of the glioblastoma xenograft in the first 25 days after injection of the cells exposed to 11PS04 (Supplementary Fig. 8B).

### Pre-treatment with 11PS04 enhances the susceptibility of glioma stem cells to temozolomide

**Figure 5:**
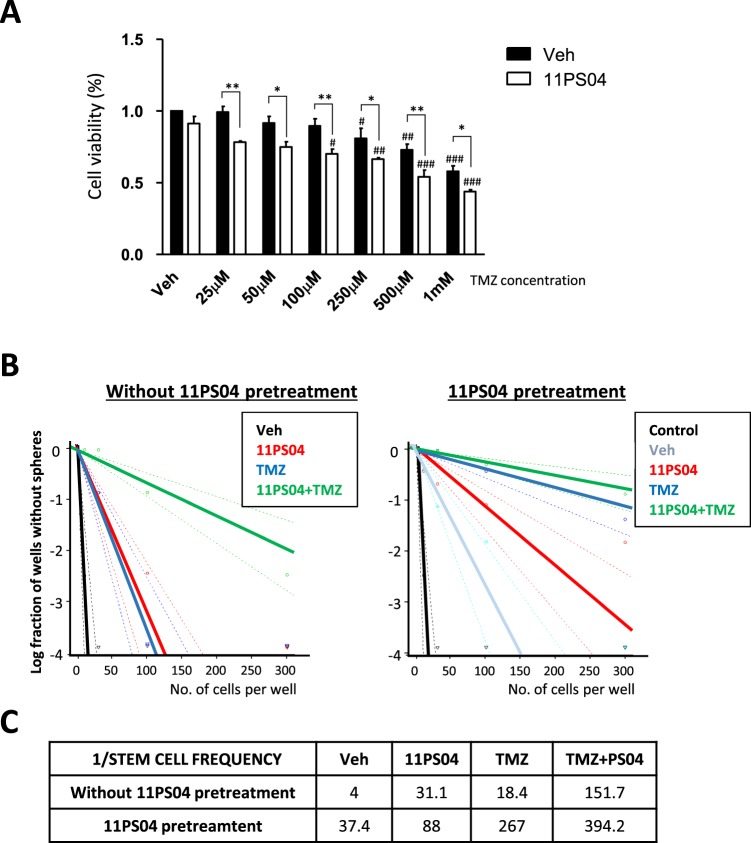
(**A**) The effect of Temozolomide (TMZ) on the viability (72 h) of U87 cells pre-treated for 48 h with 11PS04, as determined in the MTT test (n = 3): *p < 0.05, **p < 0.01, ***p < 0.001 relative to the vehicle pre-treated; ^#^p < 0.05, ^##^p < 0.01, ^###^p < 0.001 relative to vehicle cells. (**B**) Sphere frequencies from U87 single-cell suspensions are plotted relative to the number of cells seeded per well, in the presence of 11PS04 (5 μM), TMZ (100 μM) or 11PS04 (5 μM) plus TMZ (100 μM) during the formation of the spheres. Solid lines represent the frequency estimation and the dashed lines the 95% confidence intervals. The right panel shows the frequency of spheres produced by U87 cells pre-treated with 11PS04 (5 μM) or the vehicle alone, and then exposed to 11PS04 (5 μM), TMZ (100 μM) or 11PS04 (5 μM) plus TMZ (100 μM) during the formation of the spheres. The controls are the cells pre-treated and treated during sphere formation with the vehicle alone. (**C**) The frequency of initiating cancer stem cells was calculated using the ELDA platform.

A decrease in miR-21 is associated with heightened drug susceptibility and since we observed miR-21 downregulation after 11PS04 treatment, we explored whether this compound potentiates the antitumor activity of temozolomide (TMZ), currently used to clinically treat glioblastomas. U87 and rat C6 cells were grown for 48 h in the presence of 11PS04 (5 μM) under adherent conditions, after which they were exposed to different concentrations of TMZ. We found a significant reduction (*p* < 0.001) in the viability of 11PS04 pre-treated human U-87 and rat C6 cells (Fig. 5A and Supplementary Fig. 9A). In addition, while TMZ reduces the formation of spheres in the ELDA assay, pretreatment with 11PS04 made these cells dramatically more susceptible to TMZ (Fig. 5B,C and Supplementary Fig. 9). Moreover, pretreatment with 11PS04 and subsequent exposure to a combination of TMZ plus 11PS04 during their growth significantly limited the growth of the spheres, suggesting a synergistic action of 11PS04 with TMZ in sensitizing human U87 cells.

## Discussion

The attrition rates during clinical development in the pharmaceutical industry are simply too high^37–40^, a scenario that is further exacerbated if we take into account the number of compounds dropped at the pre-clinical stage. The inefficiency of drug development programs calls for urgent revision of the scientific and technological basis of innovation, particularly in relation to drug safety and efficacy. Such efforts currently focus heavily on animal models and biomarker research^40^, adopting improved and more modern strategies for drug screening, and introducing mechanistic information into the disease model to ensure a better “chain of translatability”, including adequate biomarkers and the use of 3D cultures^6–8,41^. MiRs are small RNA molecules now considered to be relevant clinical biomarkers in cancer (as reviewed in Di Leva *et al*.^19^). There has been significant research into the physiological implications of miRs, validating combinations of overlapping miRs as minimal signatures with unquestionable added value in clinical research and focusing particularly on diverse neoplasic diseases, particularly for primary diagnosis, patient stratification, therapeutic assessment and prognosis^19,42–44^. As such, miRs constitute a target for the development of therapeutic agents in many clinical circumstances^42,45^. However, despite the wealth of information generated in oncology regarding miRs in oncology, their use in drug discovery programs has only recently been contemplated, especially at the earliest preclinical stages^42,44,46–50^. We postulate here that the extensive information in oncology regarding miR signatures may help select pre-clinical candidates in drug discovery programs with high predictive validity. Nevertheless, the expensive and time-consuming methods used to analyze such signatures compromises the use of on-site miR measurement for the primary screening of libraries. The recent development of multiplexed magnetobiosensors that can profile miR signatures offers the possibility of designing automated methods to measure miRs for diagnosis, these being easier, cheaper and quicker to use than traditional techniques, with lower detection limits that require only small samples and involve no toxic reagents^51–53^.

We have access to a dual device for miR determination that was recently validated in breast cancer cell cultures and tissues, representing a novel device for the mechanistic screening of new chemical entities in libraries for which there is little information regarding their mode of action. Malignant breast cancer cell lines express substantial amounts of miR-21, an oncogenic miRNA with anti-apoptotic potential^22,26,54^. However, the mechanism underlying the effects of miR-21 on tumorigenesis remains unclear, not least because only a few targets for this miR have been verified experimentally^26,55–58^. The oncomiR miR-21 is known to exert transcriptional control over PDCD4 in malignant cells and its endogenous protein is up-regulated 3.5-fold by miR-21 inhibition^26^. PDCD4 expression is down-regulated or lost in several tumor types making it a promising molecular target for the treatment of some cancers^26,59,60^. We identified 11PS04 as a 2-phenyl-7,7a-dihydro-3a*H*-pyrano[3,*4-d*]oxazol-6(4*H*)-one derivative with an unknown mechanism of action that lowers the expression of miR-21 in MCF-7 breast cancer cells. As a result, 11PS04 upregulates PDCD4 transcription and translation, which correlates well with the observed decrease in miR-21 expression. These results show that 11PS04 potentially reverses the malignancy associated with miR-21 by restoring PDCD4, endowing this drug with a promising antitumor profile.

The re-expression of miR-21 has been associated with the acquisition of EMT in breast cancer and glioblastoma, and members of the miR-200 family and miR-205 specifically promote the generation of breast CSCs, which are associated with oncogenesis, tumor progression, metastasis and recurrence^61–65^. ZEB1 is a protein implicated in the progression of lung, colon and uterine cancers, and it is controlled by the miR-200 family and miR-205^66–68^. As expected from the expression of miR-200 family members, ZEB-1 is barely detected in epithelial cells suggesting that the downregulation of these miRs is an essential early step in tumor metastasis^20^. The EMT program itself is orchestrated by ZEB1 binding to E-boxes in the *E-cadherin* promoter, repressing its transcription and inciting metastatic outgrowth by causing carcinoma cells to enter a metastasis-initiating cell state^69–72^. Restoring miR-21 and miR-205 expression to non-tumorigenic levels by 11PS04 treatment also normalized the transcription and translation of PDCD4, ZEB-1 and E-Cadherin possibly altering the EMT program itself. However, we cannot ignore the possibility that the drug has off-targets and that deregulation of both miRs may also affect other target genes, altering downstream effectors that play a prominent role in carcinogenesis^20,63,73^. In light of the above, we conclude that 11PS04 disrupts the malignant molecular profile of two key cancer biomarkers in MCF-7 and U-87 cells, reflected by the repression of an oncomiR (miR-21) and the restoration of the tumor suppressor levels of miR-205 that both influence the behavior of CSCs and their participation in oncogenesis, tumor progression, metastasis and recurrence.

Luminal tumors have been associated with the most favorable prognosis, while Her2-overexpressing and basal-like tumors, or their surrogate triple negative tumors, have been associated with a much worse prognoses. Our aim was to assess the activity of 11PS04 against a variety of breast cancer models available in our laboratory, including one of the most frequent cell models used in breast cancer research, the Her2 negative luminal-A model (MCF-7 cells). About 10–20% of breast cancers are triple-negative breast cancers (TNBCs), a malignant disease that does not respond to hormone therapy or medicines that target HER2 protein receptors. The MDA-MB-436 cell line is a cell model currently used to study TNBCs. In addition, we were fortunate to have access to the genetically engineered Mouse Mammary Tumor Virus (MMTV)-neu+/+ mouse model, characterized by mammary gland overexpression of the human epidermal growth factor receptor (HER2) oncogene. In terms of glioblastoma, the C6 cell line is a rat model used widely in this type of cancer research, mainly due to its inherent favorable growth conditions. U-87 is also one of the most frequently used human cell model for this disease. Although many of the initial studies were carried out on C6 cells, we shifted to U-87 cells to obtain more relevant translational results.

It should be borne in mind that drug discovery targeting CSCs has not been feasible until recently, mostly due to the scarcity of these sub-populations in the tumor and their relative instability in culture^73,74^. Phenotypic 3D culturing screens that mainly measure oncospheres in suspension represent an accepted model of CSCs in breast cancer^29–32^ and glioblastoma^9,75–77^, constituting a 3D model of solid tumorigenesis (initiation, progression and recurrence)^78,79^ with higher predictive value than classical 2D growth models in culture^7,8^. In this respect, in addition to inhibiting well-accepted stem biomarkers associated to bad prognosis in these neoplasies, 11PS04 efficiently inhibits the oncosphere forming capacity of diverse cell models of breast cancer and glioblastoma, exerting a stronger inhibitory effect on oncosphere initiation than on well-organized spheres. While 11PS04 has a relevant antitumor profile in the luminal cell model of breast CSCs, its activity against Her-2/Neu tumor derived cells (MMTV-neu^+/+^), a subtype of breast cancer with a poor prognosis that affects about 30% of patients, is also noteworthy. Indeed. 11PS04 has a seven-fold stronger antitumor effect on Her-2/Neu derived stem-cells than on luminal estrogen positive MCF-7 cells, showing clear specificity for malignant tumorigenic tissue rather than normal breast tissue. The selective antineoplasic effect on Her2 over estrogen positive disease types is certainly worthy of further study, although this lies beyond the scope of this work. Nevertheless, 11PS04 does also display potent anti-neoplasic activity against well-organized spheres and against the formation of secondary spheres, which may resemble a model of tumor recurrence, reflecting the potent effect of the drug on such stage of disease progression.

Finally, definitive validation of the predictive capacity of the biosensoring platform was obtained from *in vivo* sub-epithelial xenograft studies, detecting clear and significant delays in both breast cancer and GBM tumor growth following treatment with 11PS04. The *in vivo* studies assessed the effects of 11PS04 on a tumorigenic breast cancer cell line representative of the triple negative breast cancer tumors, MDA-MB-436, and a human U-87 GBM cell model. In these studies, two significant features were maintained: (i) the same burden of malignant cells was inoculated subcutaneously into animals; and (ii) the mice were not treated with the drug after inoculation. Therefore, the data on the molecular biomarkers of cell fate and the results with the 3D CSC cultures, coupled to the significant delay in tumor growth in both neoplastic models, suggest a potential mechanistic effect of the drug on the sub-population of tumor-initiating cells (or called CSCs), a relevant result that undoubtedly merits further study.

Although chemotherapy with TMZ may restrain tumor growth for some months, tumor recurrence suggests that CSCs from these tumors persist^10,11^. There is growing interest in the therapeutic use in cancer of miRs-expression inhibitors^80–83^. The repression of miR-21 and its downstream effects, appears to have a therapeutic benefit, since decreasing it sensitizes GBM and breast cancer cells to chemotherapeutic drugs^54,84,85^. There is evidence that overexpression of miR-21 could inhibit TMZ-induced apoptosis in U87GBM cells, which highlights the possibility of that miR-21 overexpression enhace clinical resistance to TMZ chemotherapy^86^. Importantly, the synergism of 11PS04 and TMZ suggests that administering the former as an adjuvant therapeutic agent to TMZ may help improve the outcome of patients with GBM.

In summary, while some miRs have certain promiscuity in terms of their targets, minimal signatures are now being validated by clinical research in humans. The lessons learnt in clinical settings can now be translated to rational drug discovery programs addressing the appropriate physiopathological mechanisms of disease using strategies that ensure a strong “chain of translatability”. The use of multiplexed miR-magnetobiosensors with the minimal signature of only two different miRs (miR-21 and miR-205) highlights the potential of this modern technology to design predictive phenotypic screens to identify new agents that combat cancer and the persistence of epithelial CSCs. As such, we were able to show the capacity that 11PS04 merits robust protection and further preclinical development under strict regulatory rules^87^.

## Methods

### Cell culture and viability

The breast cancer cell lines, and the glioma and HeLa human carcinoma cells were cultured in DMEM (Dulbecco’s modified Eagle essential medium -DMEM: Gibco, Grand Island, NY, USA), containing 10% heat-inactivated fetal bovine serum (FBS: Gibco). Cells were grown at 37 °C in an atmosphere of 5% CO_2_ in a humidified incubator, and the viability of the MCF-7, C6 and Hela cells was measured with a CellTiter-Glo Luminescent Cell Viability Assay (Promega, Madison, WI, USA), following the manufacturer’s instructions. Briefly, a total of 10,000 cells were plated in 96-well plates and cultured for 24 h with 11PS04 (0.5 to 10 μM) in 100 μl of medium. After treatment, the plates were equilibrated to room temperature for 30 min before adding 100 μl of the Cell Titer-Glo reagent (Lysis buffer, Ultra-Glo Recombinant Luciferase, Luciferine and Mg^2+^). Luminescence was measured using a Glomax Multidetection System (Promega).

The MTT assay is a colorimetric assay to assess the metabolic activity of cells using a NAD(P)H-dependent cellular oxidoreductase reaction that under defined conditions, reflects the number of viable cells. These enzymatic reactions can reduce the tetrazolium dye MTT (3-(4,5-dimethylthiazol-2-yl)-2,5-diphenyltetrazolium bromide) to the insoluble formazan, a purple precipitate that can be measured [Stockert, J. C., Horobin, R. W., Colombo, L. L. & Blazquez-Castro, A. Tetrazolium salts and formazan products in Cell Biology: Viability assessment, fluorescence imaging, and labeling perspectives. *Acta Histochem*. **120**, 159–167, 10.1016/j.acthis.2018.02.005 (2018)]. The viability of the MCF-7, U87, C6 and CT2A cell lines was also determined after a 24 and 48 hour exposure to the drug using the MTT test (Promega), according to manufacturer’s instructions.

The viability of cells was measured using the CellTiter-Glo® Luminescent Cell Viability Assay (Promega, Madison, WI, USA). This is a quantitative method to determine the number of viable cells in culture based on the quantification of ATP, which reflects the metabolically active cells. The amount of ATP is directly proportional to the number of cells present in the culture. Subsequently, 100 μl/well of Cell Titer-Glo reagent (Lysis buffer, Ultra-Glo Recombinant Luciferase, Luciferine and Mg^2+^) was added and gently mixed for 15 minutes at room temperature (RT). The luminescence was then measured using a Glomax Multidetection System following the instructions of the supplier.

### Oncosphere culture and limiting dilution assay

Primary spheres were generated from MCF-7 and U87 cell lines, following the protocol described elsewhere^27,29^. MCF-7 and U-87 cells were detached from the plate with trypsin (Invitrogen) and plated in complete medium (DMEM/F-12 with GlutaMAX) supplemented with B27 (Gibco), 10 ng/ml epidermal growth factor (EGF: Invitrogen) and 10 ng/ml basic fibroblast growth factor (bFGF: Millipore), and they were maintained at 37 °C in 5% CO_2_. For the isolation and *in vitro* expansion of CSCs from breast tumors, the tumor tissue obtained from MMTV-HER-2/neu mice was obtained and immediately mechanically and enzymatically disaggregated, as described. Single cells were plated in bacterial plates previously coated with poly 2-hydroxyethyl methacrylate at 1,000 viable cells/ml and they were maintained in mammosphere complete media: serum-free DMEM-F12, supplemented with bFGF (2 ng/mL), EGF (200 ng/mL) and heparin (2 μg/ml) - mammosphere complete media.

For limiting dilution assays (LDAs), cells were dissociated to single cells and seeded at different dilutions in sphere growth medium (from 100 to 10 cells/well for MCF-7 cells, from 30 to 300 cells/well for U87 cells, and from 500 to 18 cells/well for Her2/Neu mouse tumor cells). The final number of spheres was quantified after 14 days, and the final data and statistical significance was obtained with the Extreme Limiting Dilution Analysis (ELDA) software (http://bioinf.wehi.edu.au/software/limdil/index.html). In the secondary sphere formation assay in the presence of 11PS04, day 7 mammospheres were exposed to the drug or the vehicle alone, and after 3 days, the spheres were dissociated with trypsin and plated in 96-well plates to perform a LDA.

### Dual detection of microRNA-21 and microRNA-205 at p19 using disposable amperometric biosensors

Amperometric measurements on the dual magnetosensor were made at room temperature according to Campuzano *et al*.^24^. Dual screen-printed carbon electrodes (SPdCEs: DRP-C1110) were purchased from DropSens and all the reagents used were of the highest available grade: Tween^®^ 20, hydroquinone (HQ), hydrogen peroxide (30%, w/v), ethylenediamine tetraacetic acid (EDTA), tris (2-carboxyethyl) phosphine hydrochloride (TCEP) and NaCl were purchased from Sigma-Aldrich; and bovine serum albumin (BSA Type VH), from Gerbu. Chitin-modified magnetic beads (Chitin-MBs: 50–70 μm, E8036S) and the p19 siRNA Binding Protein were purchased from New England Biolabs. The RNA oligonucleotides used (see Table 1) were purchased from Sigma-Aldrich.

**Table 1:** Oligonucleotides used in this study.

Oligonucleotide	Sequence (5′ → 3′)
Anti-miR-21 Probe	5′-Phos-ACAUCAGUCUGAUAAGCUA-Biotin-3′
miR-21 Target (miR-21)	5′-Phos-UAGCUUAUCAGACUGAUGU-3′
anti-miR-205 Probe	5′-Phos-AGACUCCGGUGGAAUGAAGGA-Biotin-3
miR-205 Target (miR-205)	5′-Phos-UCCUUCAUUCCACCGGAGUCU-3′

For hybridization procedure, 0.01 µM of the corresponding biotinylated anti-miR probe and the appropriate amount of the total RNA (totRNA) extracted from the samples was mixed and incubated for 30 minutes (25 °C and stirred at 250 rpm). Each dual measurement involved the preparation of two different hybridization mixtures: one supplemented with anti-miR-21 alone and another supplemented with anti-miR-205 alone. To prepare the p19-modified MBs, 2.5 μL of the Chitin-MB suspension was incubated for 20 min at 25 °C with 3 μL of the commercial p19 solution and stirred continuously (950 rpm). The supernatant was removed and the p19-bearing beads were resuspended in 20 μL of a solution containing 10 μL of the dsRNA mixture prepared as indicated above. After a 60 min incubation at 25 °C (950 rpm), the tube was placed on the magnet holding block for 3 min, the supernatant was removed, and the dsRNA-p19-MBs were resuspended in 25 μL of a 0.1 U mL^−1^ Strep-HRP solution and incubated for 15 min (25 °C). Finally, the HRP-tagged dsRNA-p19-MB conjugates were resuspended in 5 μL of 0.05 M sodium phosphate buffer saline (PBS, pH 6.0).

Dual detection at the SPdCEs was accomplished by magnetically capturing 5 μL of the re-suspended MBs modified for each miR determination on one of the two SPdC working electrodes, keeping the SPdCE in a horizontal position after placing it in the magnet holding block. The magnet holding block/SPdCE ensemble with the captured modified MBs was immersed in an electrochemical cell containing 10 mL of 0.05 M PBS (pH 6.0) and 1.0 mM HQ (prepared immediately prior to electrochemical measurement). Amperometric measurements were made at −0.20 V (relative to an Ag pseudo-reference electrode). After baseline acquisition over 60 s, 50 μL of a 0.1 M H_2_O_2_ solution was added and the current values were recorded 200 s later. The amperometric signals measured corresponded to the difference between the steady-state and the background currents, and they can be attributed to the HRP reduction of H_2_O mediated by HQ. Control tests without target miRs were included in each hybridization run to evaluate the blank signals. Unless otherwise indicated, the data given corresponded to the average of at least three replicates and the confidence intervals were calculated for α = 0.05. A new SPdCE was used for each measurement.

### RNA extraction and relative quantification of miRNA and mRNA

Cells were recovered from the plates and their total RNA was isolated using the Direct-zol RNA MiniPrep Kit (ZymoResearch). The cells were treated with the Tri Reagent and homogenized for 5 min at room temperature before using the MiniPrep Kit according to the manufacturer’s instructions. The quality and concentration of the total RNA was evaluated by measuring the absorbance at 260, 230 and 280 nm on a ND-1000 spectrophotometer (NanoDrop Technologies, Wilmington, DE, USA). In all cases, the expected 260/280 (~2.0) and 260/230 (2.0–2.2) ratios for pure RNA were obtained. To detect the two target miRs, 0.5 µg of the total RNA extracted from the cells was used for hybridization prior to measurement on the dual amperometric platform.

The qScript microRNA Quantification System (Quanta BioSciences, Inc., Gaithersburg, MD, USA) was used to assess miR-21 and miR-205 expression. In brief, cDNA was synthesized using the qScript microRNA cDNA Synthesis Kit, using 10 ng of the initial RNA per PCR reaction. The PCR conditions consisted of an initial activation at 50 °C for 2 min, followed by 40 cycles of 95 °C for 5 s and 60 °C for 30 s in a LightCycler 480 Real-Time PCR System (Roche). The *C*_*t*_ (threshold cycle) value for each primer was normalized to that of RNU6. To assess gene expression, cDNA was synthesized with the transcriptor first strand cDNA synthesis Kit (Roche) under the following PCR conditions: 40 amplification cycles of 95 °C for 10 s, 60 °C for 30 s and 72 °C for 30 s. ACTB was used as a reference gene for normalization, and the real-time PCR reactions were performed in triplicate using the PerfeCTa SYBR Green SuperMix (Quanta BioSciences) for miRNAs and the FastStart Universal SYBR Green Master (Roche) for gene expression. The expression of each gene was quantified by the relative standard curve method and the primer sequences for target genes were obtained from the Universal ProbeLibrary Assay Design Center (https://qpcr.probefinder.com/organism.jsp).

### Western blotting

Protein extracts were prepared with RIPA buffer, and equal amounts of protein were resolved by SDS-PAGE and then transferred to nitrocellulose membranes that were probed with antibodies against PDCD4 (1:5,000 dilution; Cell Signaling) and E-cadherin (1:1,000; Cell Signaling). An HRP conjugated antibody against β- actin- (1:50,000 dilution; Sigma) was used to determine the total protein loaded and densitometric analysis was performed using ImageJ software. Three independent experiments were performed for each condition.

### Xenografts

Human xenografts were injected subcutaneously into 8 to 10 week-old immunodeficient Rag2^−/−^ mice. The mice were bred and maintained at the CIB-CSIC animal facility and all animal care, handling and health monitoring was carried out in accordance with the Guidelines for the Accommodation and Care of Laboratory Animals, respecting protocols approved by the institutional animal care committee from the Comunidad de Madrid (PROEX 127/15) and the CSIC bioethics committee.

To create the xenografts, 5 × 10^5^ MDA-MB-436 cells were resuspended in 100 μL of Matrigel solution (BD Biosciences, Germany) in DMEM (1:4 v/v). The matrigel solution containing the cells was injected subcutaneously into the animals, which were monitored daily until the tumors were evident on the right flank. Once detected, the tumors were measured weekly with a caliper and their volume was calculated using the formula V = D * d2/2, where D is the long axis and d the short axis of the tumor. Finally, the animals were sacrificed and the tumors extracted.

Male 7–8 week old NOD SCID gamma (NSG) mice were injected in the dorsal flank with a total of either 10^6^ U-87 cells. The cells were treated previously for 48 h with 11PS04 (5 μM) and tumor size was measured with a caliper, every 4 days. Mice were sacrificed when the average tumor volume of the control group reached an end-point established in our ethical procedures.

### Statistical analysis

The values presented are the means ± SEM and the data from the two groups were compared with a *t* test. One-way analysis of variance (ANOVA) was used to determine the differences of the means for multiple pair-wise comparisons. The tumor growth data were analyzed using two-way ANOVA with Bonferroni post-tests: *P < 0.05, **P < 0.01, ***P < 0.001, and ****P < 0.0001. All statistical analyses were completed using Prism software.

## Supplementary information

Supplementary information

## Data Availability

The datasets generated during and or analysed during the current study are available from the corresponding author on reasonable request.
