# Supplementary material for: 11PS04 is a new chemical entity identified by microRNA-based biosensing with promising therapeutic potential against cancer stem cells
Source: Sci Rep. 2019 Aug 15;9:11916. doi: 10.1038/s41598-019-48359-y (PMC6695485; doi:10.1038/s41598-019-48359-y)
Supplement: Supplementary file 1 — Supplementary information [file 41598_2019_48359_MOESM1_ESM.pdf]

Supplementary information for:

11PS04 is a new chemical entity identified by microRNA-based biosensing with promising therapeutic potential against cancer stem cells

Tania Aguado[1], José A. Romero-Revilla[2], Rosario Granados[3], Susana Campuzano[4], Rebeca M. Torrente-Rodríguez[3], Ángel M Cuesta[1], Virginia Albiñana[1], Luisa María Botella[1], Silvia Santamaría[1], Jose A. Garcia-Sanz[1], José Manuel Pingarrón[4], Francisco Sánchez-Sancho[2,*], José-María Sánchez-Puelles[1,*]

[1] Departamento de Biomedicina Molecular, Centro de Investigaciones Biológicas, CSIC, c/ Ramiro de Maeztu 9, 28040 Madrid, Spain
[2] Instituto de Química Médica, CSIC, C/ Juan de la Cierva 3, 28006 Madrid, Spain
[3] Departamento de Anatomía Patológica, Hospital Universitario de Getafe (Spain)
[4] Departamento de Química Analítica, Facultad de Ciencias Químicas, Universidad Complutense de Madrid, E-28040
* francisco.sanchez@csic.es; * jm.spuelles@csic.es

Contents:

	Page
Scheme 1. Synthesis of compound 5 (11PS04)	S3
Experimental procedures for the synthesis of compound 5 (11PS05)	S3-S7
^{1}H-NMR and ^{13}C-NMR of compounds 1 to 5	S8-S11
X-Ray data for dinitro derivative compound	S12
Supplementary Information for the inmuno-analysis shown in Figure 2 of the article	S13
Suppplementary Figure 1	S15
Suppplementary Figure 2	S16
Suppplementary Figure 3	S17
Suppplementary Figure 4	S18
Suppplementary Figure 5	S19
Suppplementary Figure 6	S20
Suppplementary Figure 7	S21
Suppplementary Figure 8	S22
Suppplementary Figure 9	S23

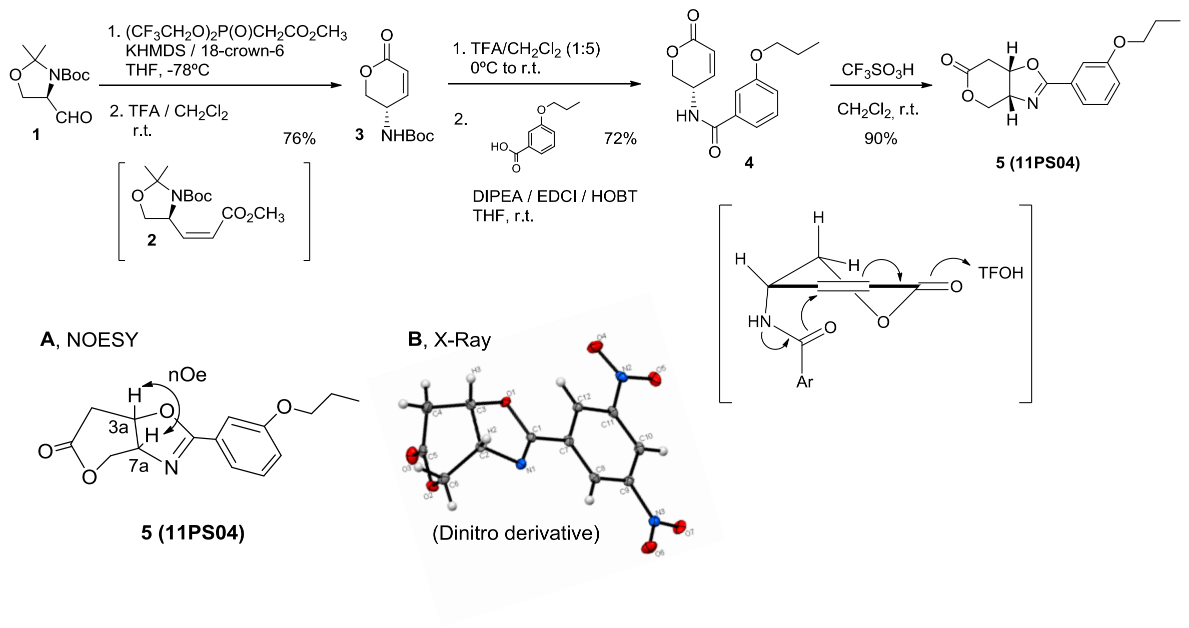

Scheme 1. Synthesis of (3a*R*,7a*S*)-2-(3-propoxyphenyl)-7,7a-dihydro-3a*H*-pyrano[3,4-*d*]oxazol-6(4*H*)-one (5; 11PS04). (**A**) Nuclear Overhauser effect correlation observed between protons H-3a and H-7a in NOESY experiments performed in compound 5 (11PS04). (**B**) X-Ray crystallographic structure of a dinitro derivative.

Experimental procedures for the synthesis of compound 5 (11PS05).

General Procedures

All reagents and starting materials were purchased from commercial suppliers and used without further purification. All the solvents used for the chemical reactions were purchased as anhydrous grade from Aldrich and they were used without further purification unless otherwise stated. Commercial grade solvents were purchased for extractions, column chromatography and thin layer chromatography (TLC). All non-aqueous reactions were performed in an argon atmosphere using standard syringe/septa techniques. In general, reactions were magnetically stirred and monitored by TLC performed on Merck TLC aluminum sheets (silica gel 60 F254). Spots were visualized with UV light (λ = 254 nm) or through staining with a phosphomolybdic acid solution or potassium permanganate solution with subsequent heating. Chromatographic purification of products was performed using

silica gel Merck-60 (230-400 mesh). Reactions at 0 °C were carried out in an ice/water bath. Reactions at −78 °C were carried out in a dry ice/acetone bath.

Nuclear magnetic resonance (NMR) spectra were recorded in deuterated solvents, as stated in each case, on Bruker AVANCE-300, Varian INOVA-300, Varian INOVA-400 and Varian INOVA-500 spectrometers. The chemical shifts measured are reported in δ (ppm) and the residual signal of the solvent was used as the internal calibration standard. All 13C NMR spectra were measured with complete proton decoupling. The multiplicity of the signals is reported as follows: s = singlet, d = doublet, t = triplet, m = multiplet, dd = doublet of doublet, dt = doublet of triplet, ddt = doublet of doublet of triplet, br = broad signal. The coupling constant *J* is reported in hertz (Hz).

HPLC-MS analyses were performed on a Waters (2695 HPLC system) apparatus, using a Sunfire^{TM} column (C18, 3.5 µm, 4.6 × 50 mm) with a solvent gradient of acetonitrile (0.08% formic acid) and H_2O (0.01% formic acid) as indicated in each case. The retention time (tr) of the peak corresponding to each product is given in minutes, and the molecular weight was determined in a quadrupolar spectrometer coupled to the HPLC system and with positive electrospray ionization (ES^+).

Elemental analysis was performed in a Heareus CHN-O-RAPID analyzer measuring the percentage of Carbon (C), Nitrogen (N), Sulfur (S) and Hydrogen (H). Optical rotation values were measured on a JASCO P-2000 Digital Polarimeter at the concentration indicated in each case (c 1 corresponds to 10 mg/mL).

(*S,Z*)-tert-Butyl 4-(3-methoxy-3-oxoprop-1-en-1-yl)-2,2-dimethyloxazolidine-3-carboxylate (2)

To a stirred solution of methyl 2-(bis(2,2,2-trifluoroethoxy)phosphoryl)acetate (1.3 mL, 6.1 mmol) in anhydrous THF (180 mL) under argon at room temperature, a solution of 18-crown-6 (5.7 g, 21.5 mmol) in anhydrous THF (4 mL) was added. The mixture was cooled to -78 ºC and stirred for 5 minutes before a 0.5 M solution of potassium

bis(trimethylsilyl)amide in toluene (12.3 mL, 6.1 mmol) was added via a syringe. Stirring was maintained for 15 minutes at this temperature and then a solution of (*R*)-*tert*-butyl 4-formyl-2,2-dimethyloxazolidine-3-carboxylate **1** (2.8 g, 5.6 mmol) in anhydrous THF (13 mL) was added. The mixture was stirred for 1 hour at -78 ºC and then diluted with a saturated aqueous solution of NH_4Cl (15 mL). This mixture was extracted with Et_2O (3x15 mL), washed with brine (2 x 20 mL), dried over anhydrous $MgSO_4$, filtered and evaporated *in vacuo*. The crude mixture was subjected to chromatography on silica gel to obtain 1.4 g (90% yield) of pure *Z*-alkene, (*S,Z*)-*tert*-butyl 4-(3-methoxy-3-oxoprop-1-en-1-yl)-2,2-dimethyloxazolidine-3-carboxylate, **2**.

^{1}H NMR (300 MHz, $CDCl_3$) δ (ppm):6.40 - 6.17 (m, 1H), 5.82 (d, 1H, *J* = 11.0 Hz), 5.38 (m, 1H), 4.32-4.17 (m, 1H), 3.76 (m, 1H), 3.70 (s, 3H), 1.61 (d, 3H, *J* = 6.0 Hz), 1.49 (d, 3H, *J* = 13.3 Hz), 1.38 (s, 9H); **^{13}C NMR** (75 MHz, $CDCl_3$) δ (ppm): 166.2, 152.3, 95.3, 80.3,125.6, 122.5, 68.2, 59.2, 51.9, 28.7, 27.0, 26.8; **HPLC- MS (ES$^+$)**: Gradient MeCN/H_2O 40:60 to 100:0 (5 min), tr: 3.98 min, $[M+H]^+$ = 286.3; **EA** calculated for $C_{14}H_{23}NO_5$: C 58.93, H 8.12, N 4.91 obtained C 59.04, H 8.22, N 5.06; **Optical rotation**: $[\alpha]_D$=-28 (c =1, $CHCl_3$).

(*S*)-*tert*-Butyl (6-oxo-3,6-dihydro-2*H*-pyran-3-yl)carbamate (3)

Trifluoroacetic acid (TFA: 0.5 mL, 6 mmol) was added to a stirred solution of (*S,Z*)-*tert*-butyl 4-(3-methoxy-3-oxoprop-1-en-1-yl)-2,2-dimethyloxazolidine-3-carboxylate via a syringe, **2**, (850 mg, 2.95 mmol) in anhydrous CH_2Cl_2 (35 mL) under an argon atmosphere at room temperature. The mixture was stirred overnight and then diluted with dichloromethane, and this mixture was washed with a saturated aqueous solution of $NaHCO_3$ until a basic pH was obtained. The organic phase was washed with brine, dried over anhydrous $MgSO_4$, filtered and evaporated *in vacuo* to obtain 508mg of lactone **3**, (*S*)-*tert*-Butyl (6-oxo-3,6-dihydro-2*H*-pyran-3-yl)carbamate (80% yield).

^{1}H NMR (300 MHz, $CDCl_3$) δ (ppm): 6.88 (dd, 1H, *J* = 9.5, 4.6 Hz), 6.09 (dd, 1H, *J* = 9.8, 0.9 Hz), 4.77 (broad s, 1H), 4.53 - 4.32 (m, 3H), 1.45 (s, 9H); **^{13}C NMR** (75 MHz, $CDCl_3$) δ (ppm): 162.5, 155.7, 144.3, 122.9, 79.2, 70.5, 42.7, 28.2. **HPLC-MS (ES^{+}):** Gradient MeCN/H_2O 10:90 to 100:0 (5 min), tr: 4.7 min, $[M+23]^{+}$ = 236. **EA** calculated for $C_{10}H_{15}NO_4$: C 56.33, H 7.09, N 6.57 obtained C 56.32, H 7.10, N 6.61; **Optical rotation**: $[\alpha]_D$ = +113 (c = 1.15, $CHCl_3$) Lit [63]. $[\alpha]_D$ = +105 (c = 1.06, $CHCl_3$).

(*S*)-3-Propoxy-*N*-(6-oxo-3,6-dihydro-2*H*-pyran-3-yl)benzamide (4)

A solution of lactone **3** (200 mg, 0.9 mmol) in anhydrous dichloromethane (8 mL) was cooled to 0 ºC in an argon atmosphere and TFA (1.52 mL, 19.7 mmol) was then added to the solution at room temperature. The mixture was stirred at 0 ºC for 5 minutes, removed from the bath, left to reach room temperature slowly and then stirred for 1 hour. When the reaction was completed, the mixture was diluted with dichloromethane and the solvent evaporated at reduced pressure (x4) in order to remove the remaining TFA.

The unprotected lactone was dissolved in anhydrous dichloromethane under argon and at room temperature. Then, *N,N*-diisopropylethylamine (DIPEA; 0.5 mL, 2.8 mmol) was added via a syringe, followed by 3-propoxybenzoic acid (202 mg, 1.1 mmol), EDCI (320 mg, 1.7 mmol) and HOBt (151 mg, 1.1 mmol) in anhydrous THF (9 mL). When the reaction was complete, the mixture was treated with a saturated aqueous solution of NH_4Cl (5 mL) and extracted with EtOAc (3 x 10 mL). The organic layer was washed with saturated solution of NaCl (2 x 10 mL), dried over $MgSO_4$, filtered and concentrated to dryness. The crude product was purified by silica gel chromatography (hexane:EtOAc 5:1 to 3:1) to obtain the desired compound, **4**, as a white solid (186mg, 72% yield).

^{1}H NMR (400 MHz, $CDCl_3$) δ (ppm): 7.70 (d, *J* = 8.3 Hz, 1H), 7.41 – 7.35 (m, 2H), 7.25 (t, *J* = 8.1 Hz, 1H), 7.00 (ddd, *J* = 8.2, 2.5, 1.1 Hz, 1H), 6.92 (ddd, *J* = 9.7, 5.3, 1.2 Hz, 1H), 6.05 (dd, *J* = 9.7, 1.1 Hz, 1H), 5.01 – 4.88 (m, 1H), 4.51 (dd, *J* = 11.9, 4.1 Hz, 1H), 4.44 (ddd, *J* = 11.9, 3.3, 1.3 Hz, 1H), 3.87 (t, *J* = 6.6 Hz, 2H), 1.75 (q, *J* = 6.9 Hz, 2H), 0.98 (t, *J*

= 7.4 Hz, 3H). **^{13}C NMR** (101 MHz, $CDCl_3$) δ (ppm): 167.4, 162.8, 159.8, 143.9, 134.8, 129.9, 123.6, 119.1, 119.0, 113.6, 70.7, 70.1, 42.1, 22.7, 10.6. **HPLC-MS (ES^+)**: Gradient MeCN/H_2O 10:90 to 100:0 (5 min), tr: 4.2 min, $[M+1]^+$ = 276. **E.A.** calculated for $C_{15}H_{17}NO_4$: C 65.44, H 6.22, N 5.09 found C 65.18, H 6.09, N 4.90.

(3a*R*,7a*S*)-2-(3-Propoxyphenyl)-7,7a-dihydro-3a*H*-pyrano[3,4-*d*]oxazol-6(4*H*)-one (5; 11PS04)

A solution of compound **4** (155mg, 0.56mmol) was prepared in anhydrous dichloromethane under argon and at 0 ºC. Triflic acid (TfOH, 0.25ml, 2.77mmol) was added to this via a syringe and the mixture was stirred at room temperature for 2 hours. When the reaction was complete, the mixture was diluted with dichloromethane, the pH was adjusted with a saturated aqueous solution of K_2CO_3 and it was extracted with dichloromethane. The organic extract was dried with anhydrous $MgSO_4$ and filtered before removal of the solvent. The crude product was purified on a silica gel column eluting with mixtures of hexane/AcOEt to obtain the title compound, **5d**, as a white solid (139mg, 90% yield).

^{1}H RMN (300 MHz, $CDCl_3$) δ (ppm): 7.47 (m, 1H), 7.41 (m, 1H), 7.35 – 7.25 (m, 1H), 7.03 (ddd, *J* = 8.3, 2.7, 1.0 Hz, 1H), 5.24 (ddd, *J* = 9.9, 4.3, 2.7 Hz, 1H), 4.64 (ddd, *J* = 10.0, 3.1, 1.9 Hz, 1H), 4.59 (dd, *J* = 12.3, 2.0 Hz, 1H), 4.42 (dd, *J* = 12.4, 3.2 Hz, 1H), 3.96 (t, *J* = 8.0, 2H), 3.10 (dd, *J* = 16.2, 2.8 Hz, 1H), 2.80 (dd, *J* = 16.2, 4.2 Hz, 1H), 1.82 (m, 2H), 1.06 (t, *J* = 8.0, 3H). **^{13}C RMN** (75 MHz, $CDCl_3$) δ (ppm): 168.8, 165.4, 159.4, 129.6, 127.9, 121.0, 119.4, 114.2, 75.3, 70.0, 68.9, 64.5, 34.5, 22.7, 10.6. **HPLC-MS (ES^+):** Gradient MeCN/H_2O 10:90 to 100:0 (5 min), tr: 4.3 min, $[M+H]^+$ =277.3. **E.A.** calculated for $C_{15}H_{17}NO_4$: C, 65.44%; H, 6.22%; N, 5.09%; obtained: C, 65.28%; H, 6.27%; N, 5.23%. **Optical rotation:** $[\alpha]_D$ = -72 (c = 1, $CHCl_3$).

^{1}H-NMR and ^{13}C-NMR of compounds 1 to 5.

^{1}H-NMR (400MHz, C_6D_6, 70°C)

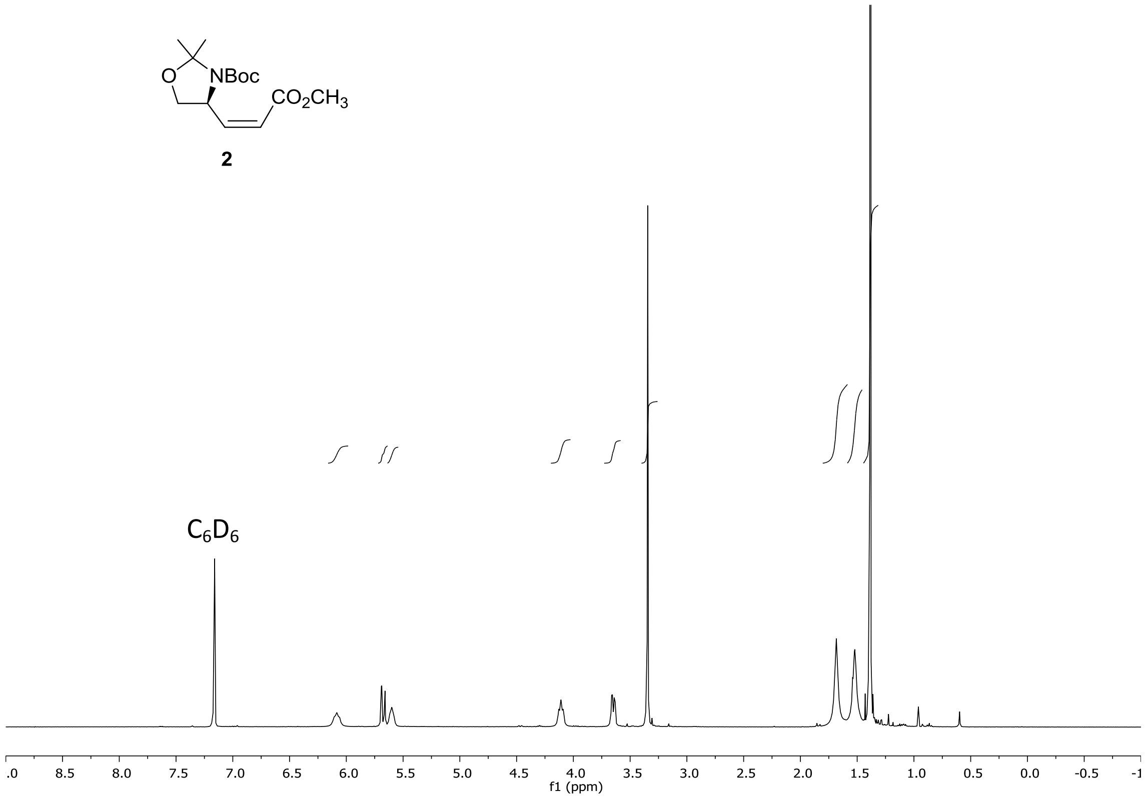

^{13}C-NMR (100MHz, C_6D_6, 70°C)

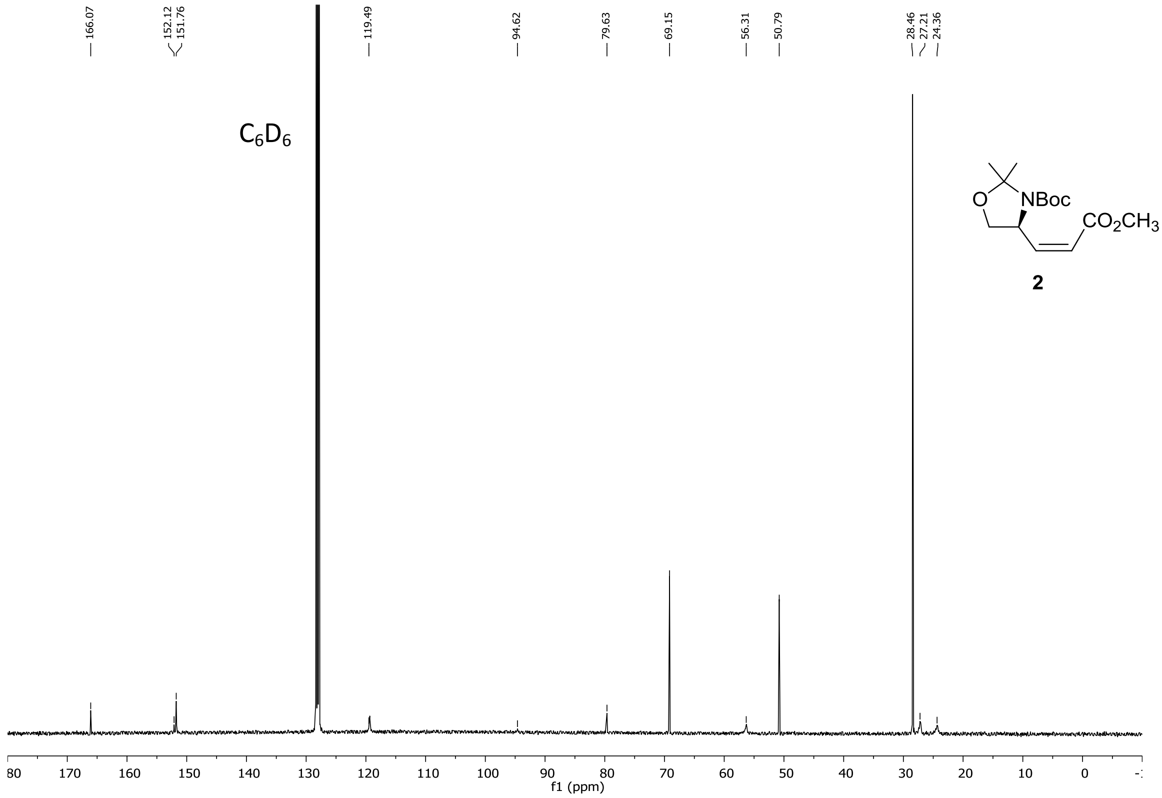

^{1}H-NMR (300MHz, $CDCl_3$)

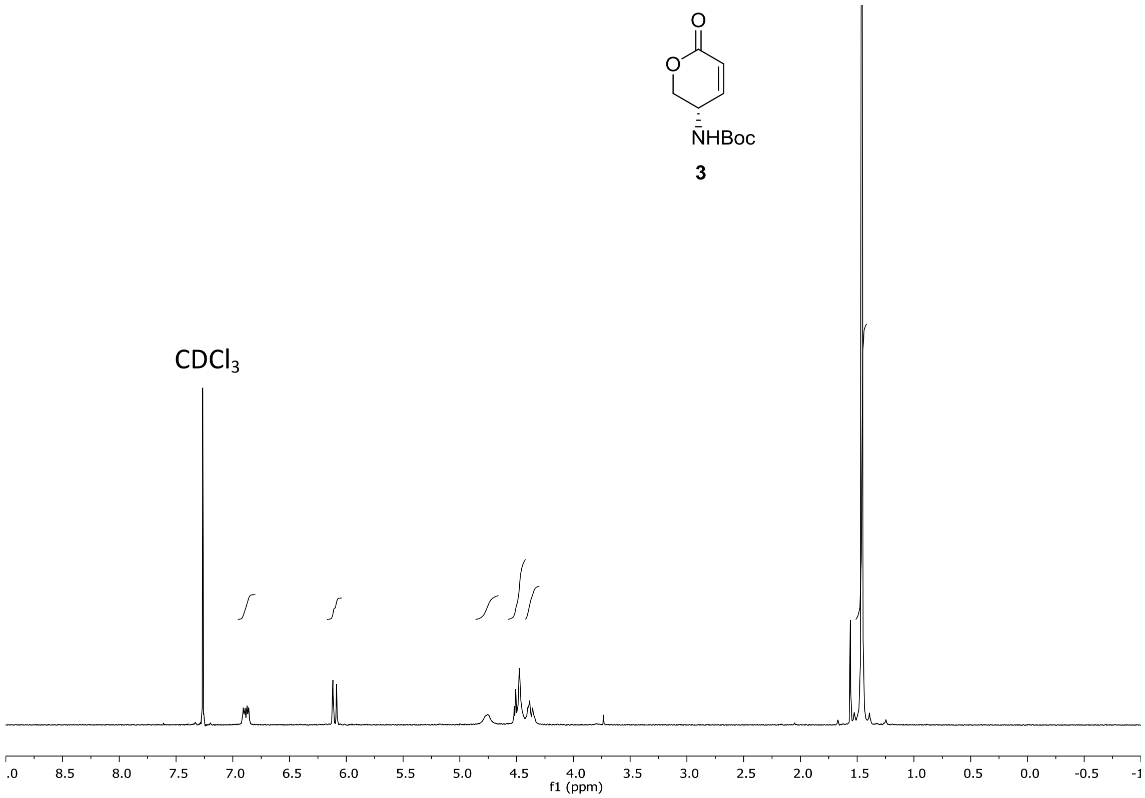

^{13}C-NMR (100MHz, $CDCl_3$)

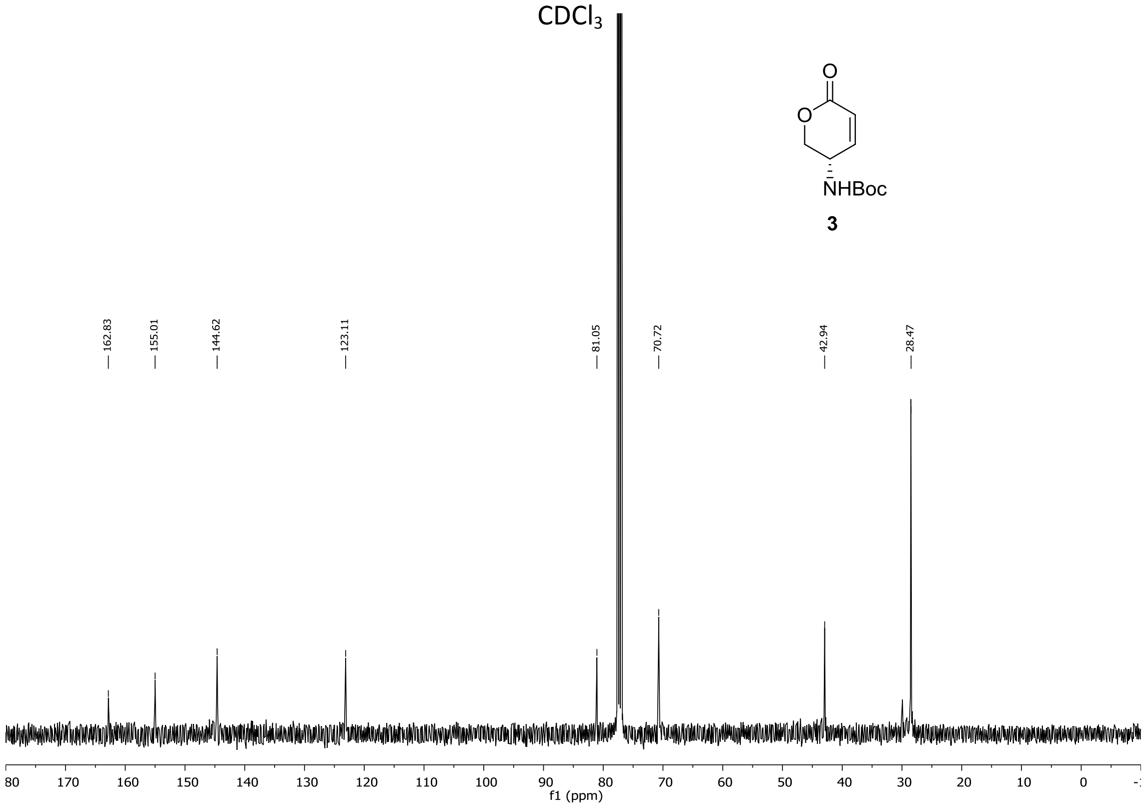

^{1}H-NMR (400MHz, $CDCl_3$)

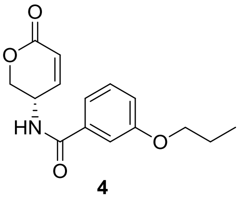

4

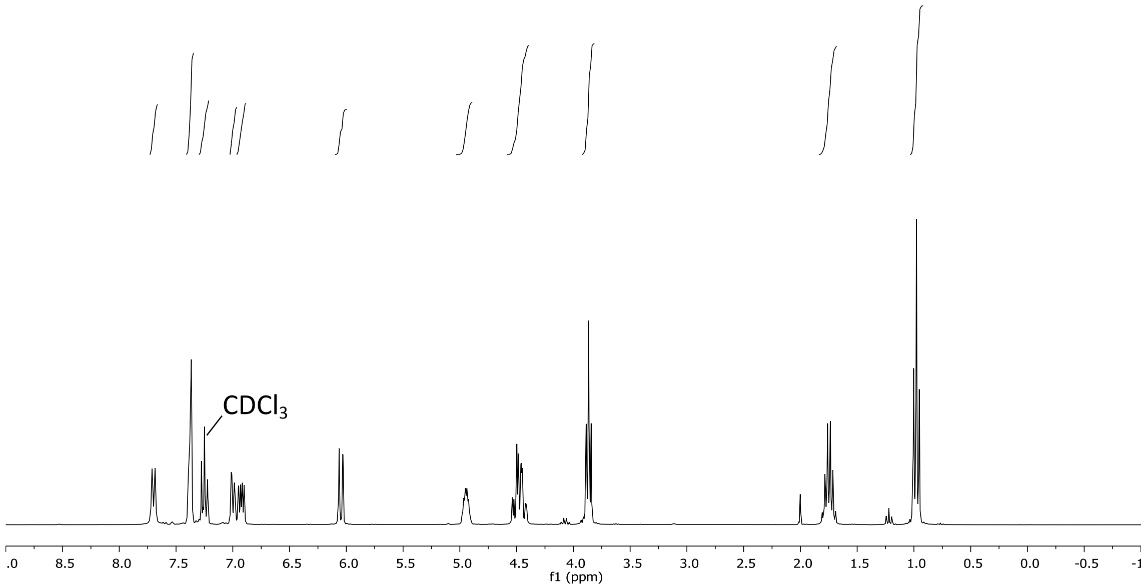

^{13}C-NMR (100MHz, $CDCl_3$)

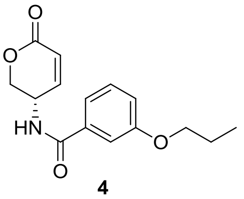

4

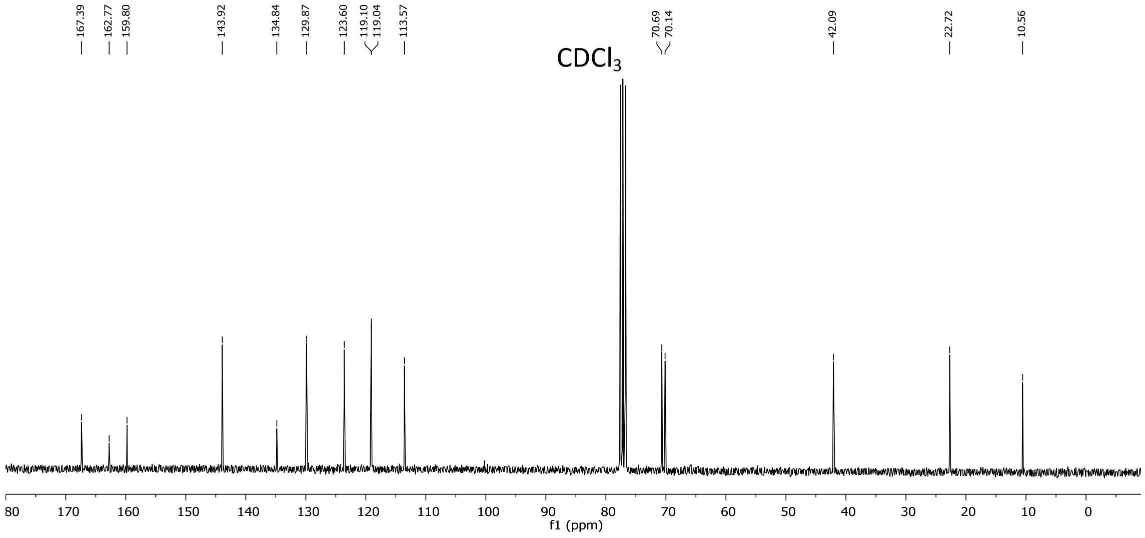

^{1}H-NMR (300MHz, $CDCl_3$)

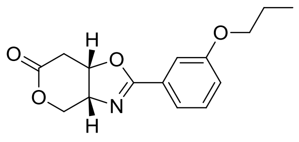

5 (11PS04)

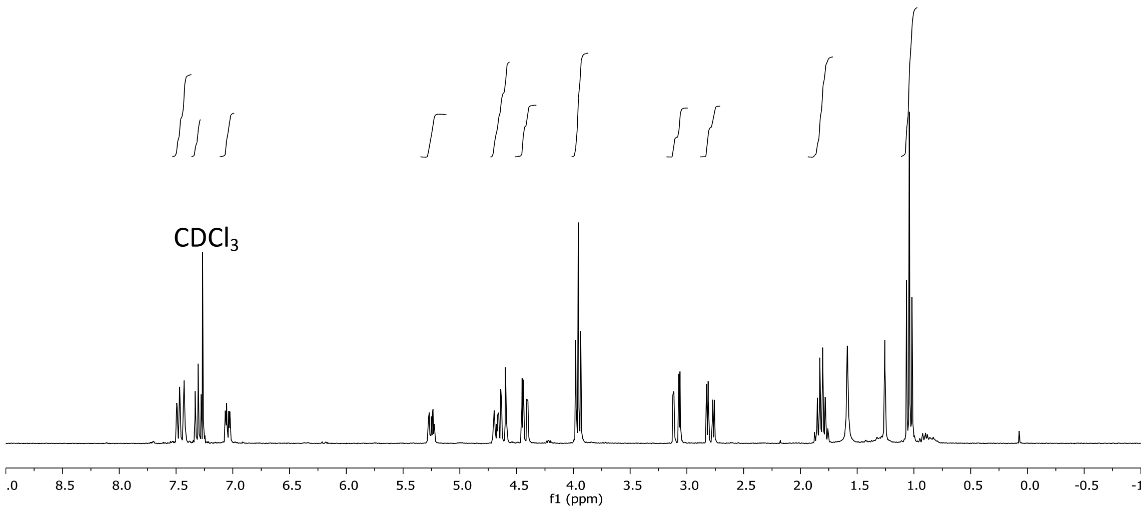

^{13}C-NMR (75MHz, $CDCl_3$)

$CDCl_3$

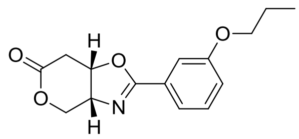

5 (11PS04)

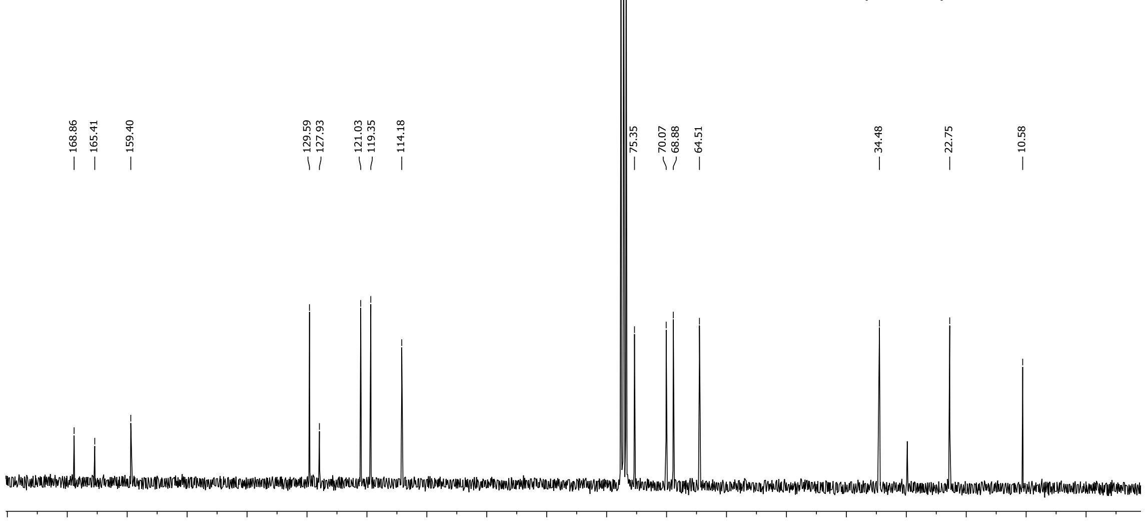

X-Ray data for dinitro derivative compound.

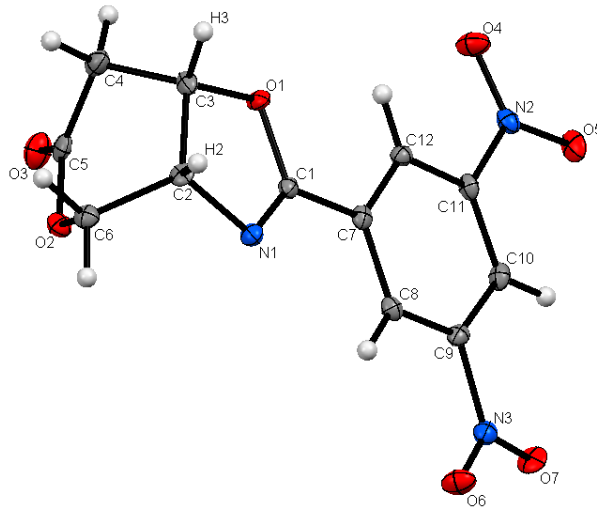

Crystal data

Empirical formula: $C_{12}H_9N_3O_7$

Formula weight*:* 307.22

Symmetry cell: Orthorhombic

Symmetric space group name: *P*2(1)2(1)2(1)

Cell volume: 1291.4(8) Å^3

Cell formula units: *Z* = 4

Cell length a: 6.337(2) Å α= 90°

Cell length b: 9.033(4) Å β= 90°

Cell length c: 22.559(6) Å γ = 90°

Cell measurement temperature: 100(2) K

Cell measurement reflections: 3225

Cell measurement theta (θ): 1.81 - 27.95°

Mo *K*α radiation, λ = 0.71073 Å

Absorption coefficient: 0.133 mm^{-1}

Density (calculated) = 1.580 Mg/m^3

Crystal F(000): 632

Crystal size (max, mid, min): 0.34 x 0.23 x 0.18 mm^3

Max. and min. transmission: 0.9764 and 0.9561

Goodness-of-fit on F^2: 1.160

Final R indices [I>2Σ(I)]: R1 = 0.0282, wR2 = 0.0808

R indices (all data): R1 = 0.0323, wR2 = 0.1058

Absolute structure parameter: -0.1(13)

Largest diff. peak and hole; 0.209 and -0.230 e.Å^{-3}

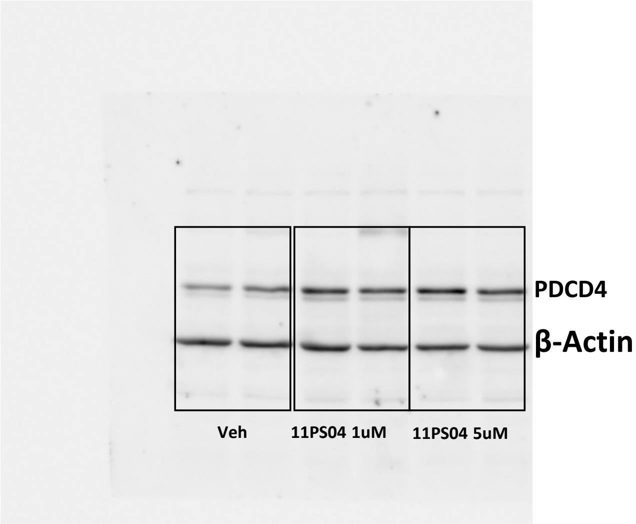

Supporting information corresponding to the inmuno-analysis shown in Figure 2 (1 of 4): Image corresponding to the direct capture of the Western blot corresponding to the extracts from MCF7 breast cancer cell line treated with 11PS04 and using PDCD4 and β-Actin antibodies.

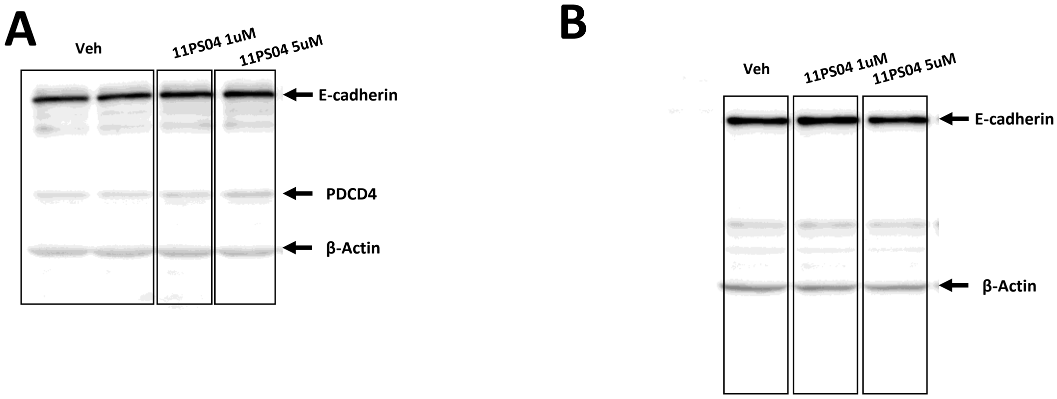

Supporting information of the inmuno-analysis shown in Figure 2 (2 of 4): Image corresponding to the direct capture of the Western blot of the extracts from MCF7 breast cell line treated with 11PS04 and using PDCD4, E-cadherin and β-Actin antibodies: A) Western blot analysis of extracts from MCF7 treated with 11PS04 using PDCD4, E-cadherine and β-Actin antibodies. B) Western blot analysis of extracts from MCF7 treated with 11PS04, using the E cadherine and β-Actin antibodies.

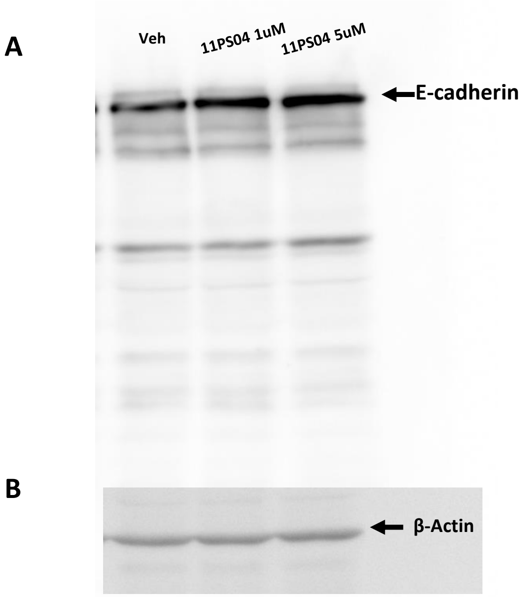

Supporting information of the inmuno-analysis shown in Figure 2 (3 of 4): Image corresponding to the direct capture of the Western blot of the extracts from MCF7 breast cell line treated with 11PS04 and using E-cadherin and β-Actin antibodies: A) Western blot analysis of extracts from MCF7 treated with 11PS04 using E-cadherine antibody. B) Western blot analysis of extracts from MCF7 treated with 11PS04 corresponding to membrane showed in A) but using the β-Actin antibody.

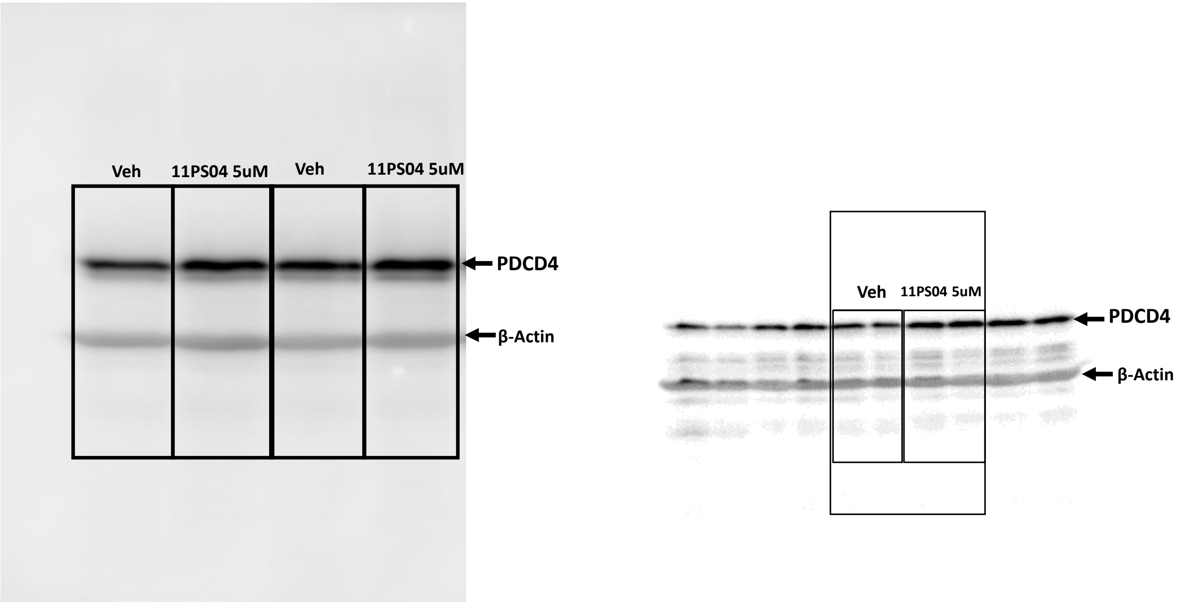

Supporting information of the inmuno-analysis shown in Figure 2 (4 of 4): Images corresponding to three different experiments: the direct capture of the Western blots of the corresponding extracts from human glioblastoma U-87 cell line treated with 11PS04 and using PDC4 and β-Actin antibodies.

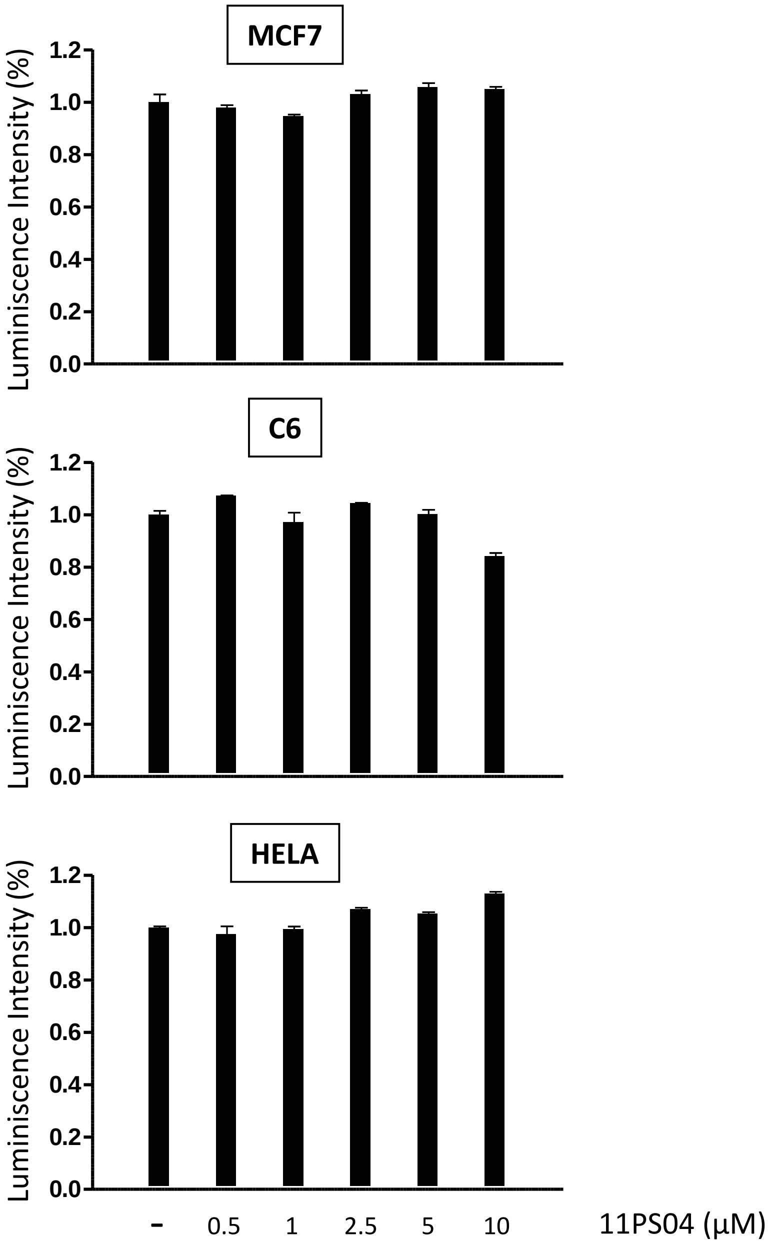

Suppplementary Figure 1. Dose-reponse effect of 11PS04 on the viability (72h) of MCF-7, C6 and HeLa cancer cell models. Cells were grown at 37 °C in an atmosphere of 5% CO_2 in a humidified incubator, pre-treated with 11PS04 during 48 h and the viability was measured with a CellTiter-Glo Luminescent Cell Viability Assay (mean ± SEM; n = 3).

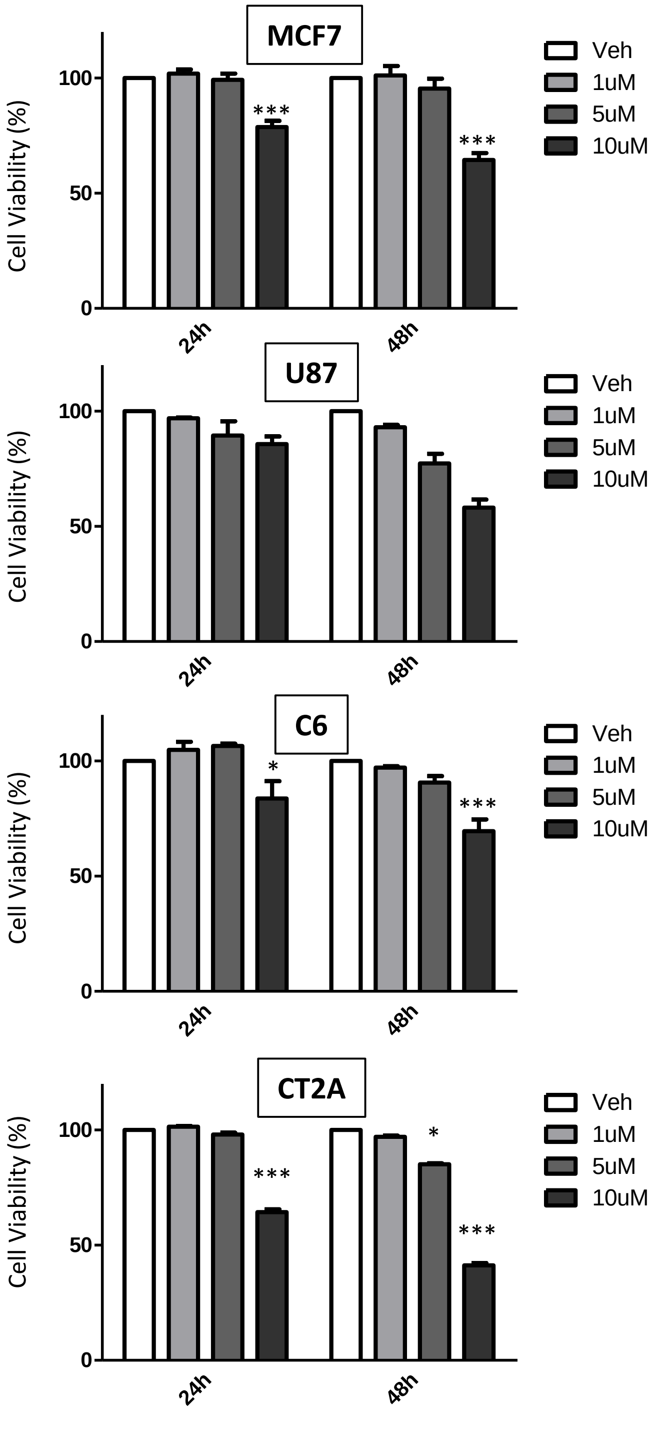

Supplementary Figure 2. Effect of 11PS04 on the viability (24h and 48h) of MCF7, U87, C6 and CT2A cells as determined by the MTT test (mean ± SEM; n = 3.*p< 0.05, **p< 0.01, ***p< 0.001).

MDA-436

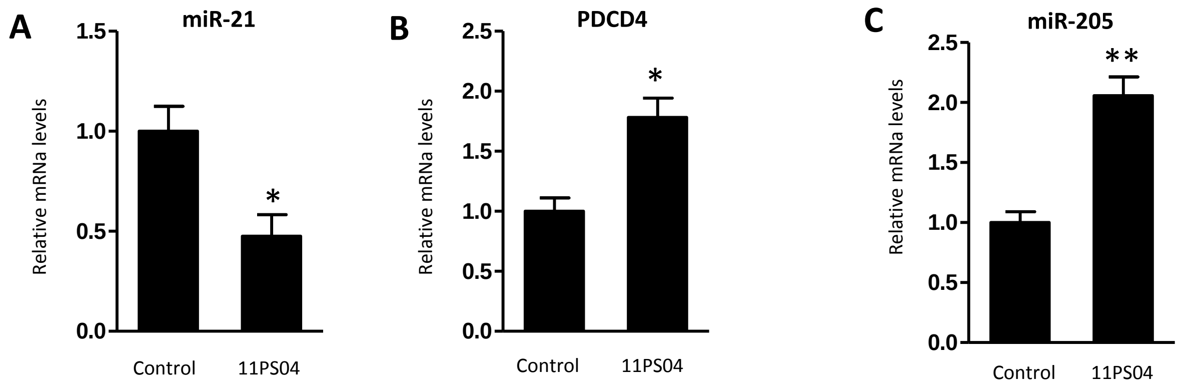

Supplementary Figure 3. 11PS04 regulates the expression of miR-21 and miR-205 in MDA-436 cell line. 11PS04 treatments led to a dose-dependent regulation of the transcriptional expression of miR-21 (A), PDCD4 (B), miR-205 (C), Differences were statistically significant according to the Student's t-test (mean ± SEM; n = 3.*p< 0.05, **p< 0.01, ***p< 0.001).

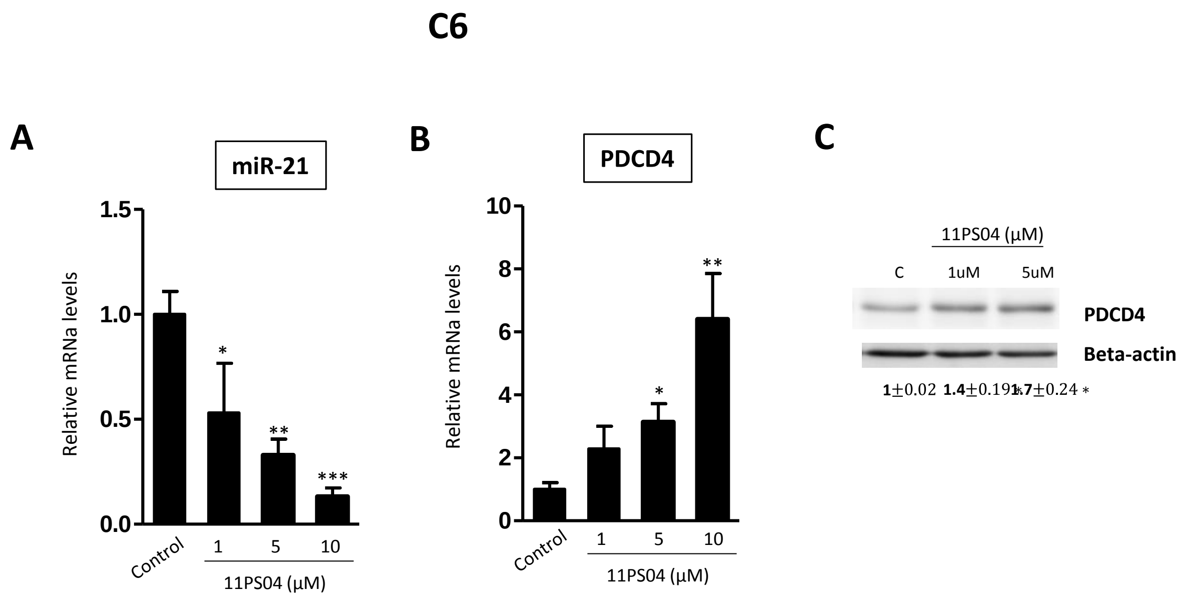

Supplementary Figure 4. 11PS04 regulates the expression of miR-21 and downstream target gene PDCD4 in rat C6 cell line. 11PS04 treatments led to a dose-dependent regulation of the transcriptional expression of miR-21 (A), and PDCD4 (B). The transcription levels were compared with the endogenous control of ACTB ribosomal RNA. Expression of the target protein PDCD4 (C) were measured by Western blot analysis of extracts from C6 treated cultures. Differences were statistically significant according to the Student's t-test (mean ± SEM; n >3.*p< 0.05, **p< 0.01, ***p< 0.001).

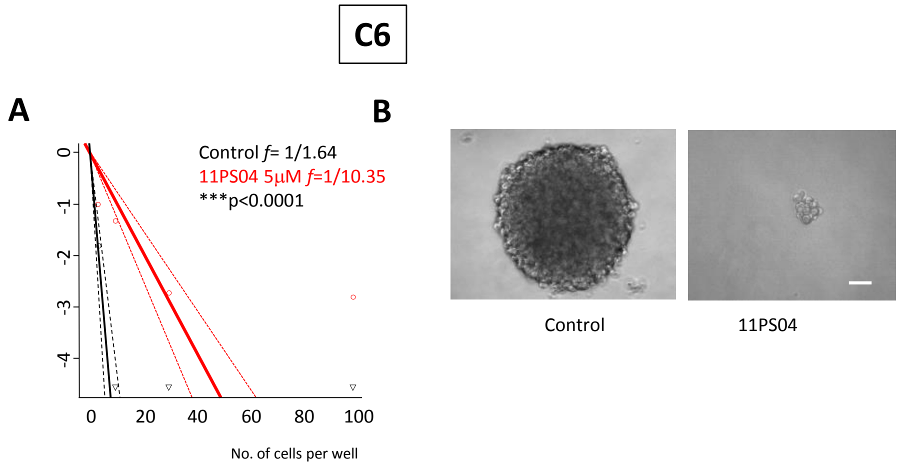

Supplementary Figure 5. 11PS04 decreases glioma C6 sphere-forming capabilities. (A) Sphere frequencies from C6 single-cell suspensions are plotted vs the number seeded per wells, showing a decreased sphere formation under 11PS04 treatments during the forming process. Solid lines represent the frequency estimate; and the nonsolid lines, 95% confidence intervals. Frequency of initiating cancer stem cells was calculated using the ELDA platform. The difference in mammosphere frequency among 11PS04 treatment and control group was significant, with a P value < 0.001. (B) Representative phase-contrast images of spheres formed at day 15. (Scale bar 40 µm.).

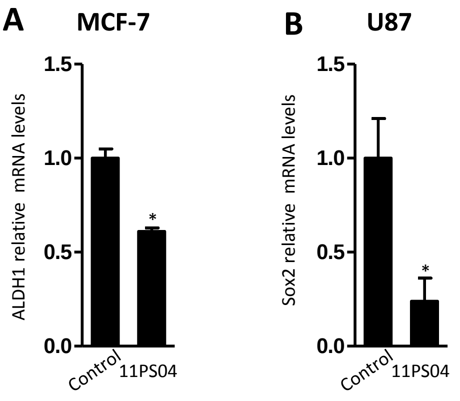

Supplementary Figure 6. 11PS04 decreases the expression of cancer stem cell markers. (A) MCF7 growing mammospheres were exposed 11PS04 (5µM) for 2 days, and ALDH1 mRNA levels were determined by RT-qPCR . (B) 11PS04 treatments (5µM) of during gliosphere formation process, through 2 days, regulate the transcriptional expression of Sox2. The transcription levels were compared with the endogenous control of ACTB ribosomal RNA (mean ± SEM; *n* = 3; ***P* < 0.01)

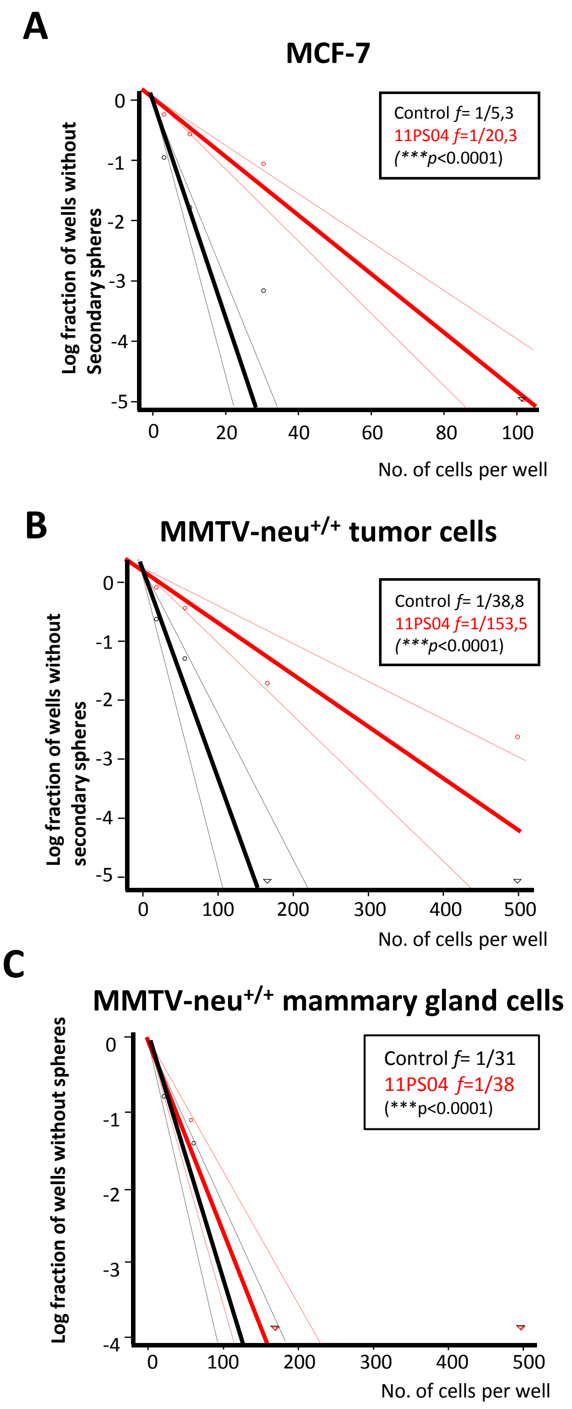

Supplementary Figure 7. (A) Sphere frequencies in secondary sphere-forming capabilities from MCF-7. (B) MMTV-neu$^{+/+}$mice tumorspheres. Decrease in the number of secondary spheres upon 11PS04 treatment starting on day 7 on the formed mamospheres. (C) Sphere frequencies from mammary glands from MMTV-neu$^{+/+}$mice, plotted vs the number seeded per wells, showing the same sphere formation capacity under 11PS04 treatments during the forming process. The frequencies of sphere formation were measured by the extreme limited dilution analysis (ELDA).Solid lines represent the frequency estimate; and the nonsolid lines, 95% confidence intervals. Frequency of initiating cancer stem cells was calculated using the ELDA platform.

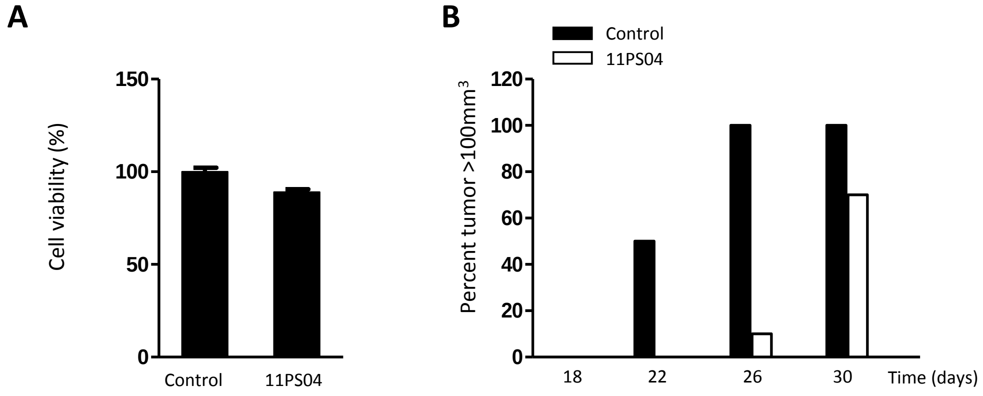

Supplementary Figure 8. (A) After injecting the U87 cells in the mice, they were plated in P96 plates. After 72h, a MTT was carried out without significant differences in the viability of those pretreated with 11PS04 with respect to cells treated with DMSO (mean ± SEM; n >3). **(B)** The graph shows the percentage of mice with tumors greater than 100mm3 at different times.

A

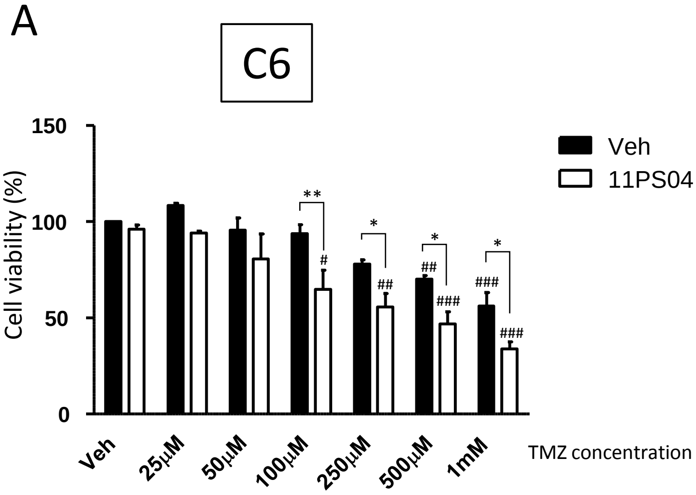

B

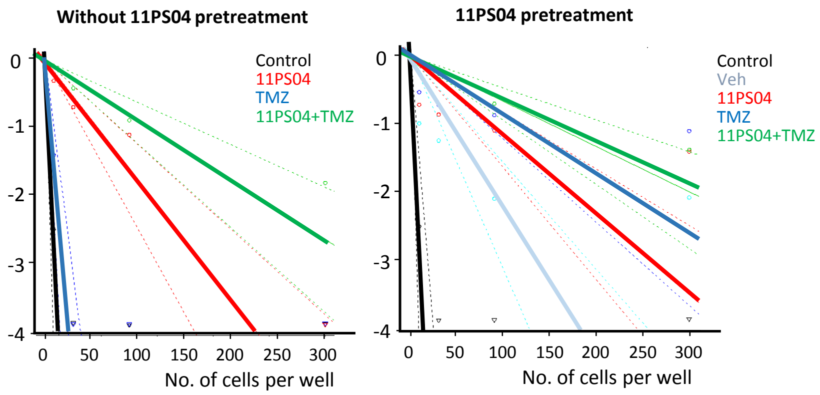

C

1/STEM CELL FREQUENCY	Veh	11PS04	TMZ	TMZ+PS04
**Without pretreatment**	1.2	18.9	2.04	38.1
**11PS04 pretreamtent**	15.1	29.9	38.6	59.4

Supplementary Figure 9. (A) Effect of Temozolomide (TMZ) on the viability (72h) of C6 pre-treated 48h with 11PS04 as determined by the MTT test n=3; *p< 0.05, **p< 0.01, ***p< 0.001 from vehicle pre-treated; #p< 0.05, ##p< 0.01, ###p< 0.001 from vehicle cells **(B)** Sphere frequencies from U87 single-cell suspensions are plotted versus the number seeded per wells, under 11PS04 (5µM), TMZ (100µM) or 11PS04 (5µM) plus TMZ (100µM) treatments during the forming process. Solid lines represent the frequency estimate; and the nonsolid lines, 95% confidence intervals. Right panel show spheres frequencys from C6 11PS04 (5µM) pre-treated and Veh, 11PS04 (5µM), TMZ (100µM) or 11PS04 (5µM) plus TMZ (100µM) treatments during the forming process. Control represents cells no preteated neither treated during sphere formation. **(C)** Frequency of initiating cancer stem cells was calculated using the ELDA platform.
